# Single-step synthesis and interface tuning of core–shell metal–organic framework nanoparticles†

**DOI:** 10.1039/d0sc03940c

**Published:** 2021-02-09

**Authors:** Kieran W. P. Orr, Sean M. Collins, Emily M. Reynolds, Frank Nightingale, Hanna L. B. Boström, Simon J. Cassidy, Daniel M. Dawson, Sharon E. Ashbrook, Oxana V. Magdysyuk, Paul A. Midgley, Andrew L. Goodwin, Hamish H.-M. Yeung

**Affiliations:** Inorganic Chemistry Laboratory, University of Oxford South Parks Road Oxford OX1 3QR UK; Cavendish Laboratory, University of Cambridge 19 JJ Thomson Avenue Cambridge CB3 0HE UK; Department of Materials Science and Metallurgy, University of Cambridge 27 Charles Babbage Road Cambridge CB3 0FS UK; School of Chemical and Process Engineering & School of Chemistry, University of Leeds LS2 9JT UK; ISIS Neutron and Muon Facility, STFC Rutherford Appleton Laboratory Chilton Didcot Oxon, OX11 0QX UK; Max Planck Institute for Solid State Research Heisenbergstrasse 1 70569 Stuttgart Germany; Department of Chemistry, University of St Andrews North Haugh St Andrews KY16 9ST UK; Diamond Light Source Ltd., Harwell Science and Innovation Campus Didcot OX11 0DE UK; School of Chemistry, University of Birmingham Haworth Building, Edgbaston Birmingham B15 2TT UK h.yeung@bham.ac.uk +44 (0)121 414 8811

## Abstract

Control over the spatial distribution of components in metal–organic frameworks has potential to unlock improved performance and new behaviour in separations, sensing and catalysis. We report an unprecedented single-step synthesis of multi-component metal–organic framework (MOF) nanoparticles based on the canonical ZIF-8 (Zn) system and its Cd analogue, which form with a core–shell structure whose internal interface can be systematically tuned. We use scanning transmission electron microscopy, X-ray energy dispersive spectroscopy and a new composition gradient model to fit high-resolution X-ray diffraction data to show how core–shell composition and interface characteristics are intricately controlled by synthesis temperature and reaction composition. Particle formation is investigated by *in situ* X-ray diffraction, which reveals that the spatial distribution of components evolves with time and is determined by the interplay of phase stability, crystallisation kinetics and diffusion. This work opens up new possibilities for the control and characterisation of functionality, component distribution and interfaces in MOF-based materials.

## Abstract 2

Core–shell metal–organic framework nanoparticles have been synthesised in which the internal interface and distribution of components is found to be highly tunable using simple variations in reaction conditions.

## Graphical abstract

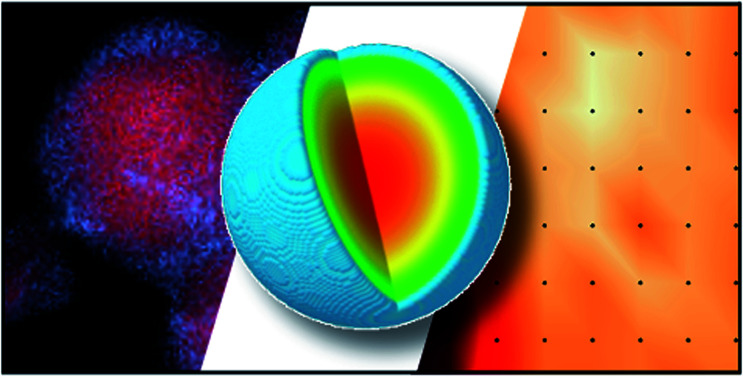

## 1 Introduction

Combining different components on a single crystalline lattice gives rise to a wealth of cooperative behaviour and tunability in materials as diverse as ferroelectrics, superconductors, photovoltaics, engineering alloys, and catalysts. In recent years, mixed-component metal–organic frameworks (MOFs), sometimes referred to as multivariate MOFs,^1,2^ containing as many as ten metals^3^ or eight organic linkers^4^ in a single phase have been reported. On the other hand, multi-component epitaxial thin films^5,6^ or particles^7–10^ can exhibit phase separation when synthesis conditions promote temporal separation of the formation of the different phases. For example, on-particle MOF-on-MOF coatings^7,11,12^ and core–shell structures^8,13^ may be synthesised in multiple steps, typically when differences in lattice parameters are small, which enables epitaxial growth of one phase on the other. Alternatively, core–shell MOF particles with micron dimensions^9^ or nanoparticles^14^ have been synthesised in a single step when growth kinetics are significantly different. When multiple components are combined, their spatial distribution is critical to the function of the material as a whole.^7,9,15–21^ Emerging MOF applications, such as smart molecular sorting, photocatalysis, enzymatic tandem reactions and electronic devices, will require even more precise control over spatial distribution, in order to tune nano- and mesoscopic diffusional properties.^22–26^ However, the necessary synthetic control that will underpin such developments has largely been overlooked in bulk materials. This is in part due to the inherent challenge of nanostructure characterisation^27^ but also the limitations in understanding of the MOF formation process.^28,29^

We report the first single-step synthesis of multi-component core–shell MOF nanoparticles with an internal interface with tunable diffuseness and position, using the Zn-based ZIF-8 system^30^ and its Cd analogue.^31^ This system has been shown to form solid solutions with the formula Zn_1−*x*_Cd_*x*_(mIm)_2_ (mIm = 2-methylimidazolate)^32,33^ and similar metal substitutions have been shown to affect pore apertures, phase changes and gas sorption characteristics.^20,32,34^ Formation of core–shell structures with large differences in pore diameter and aperture (in this case, 11.6 Å *vs.* 14.2 Å and 3.4 Å *vs.* 3.9 Å, respectively) may lead to tunable adsorption properties of the material, owing to the different capacities and/or size selectivities of the components.^7^ We use scanning transmission electron microscopy X-ray energy dispersive spectroscopy (STEMEDS) to show that the nanoparticles consist of a Cd-rich core surrounded by a Zn-rich shell, whose internal structure depends on synthesis conditions. By implementing a newly-devised composition gradient model to analyse 99 high-resolution synchrotron X-ray diffraction (XRD) datasets, we show that not only the compositions of core and shell but also the position and diffuseness of the core–shell interface can be tuned *via* the synthetic variables of reaction temperature and reactant ratio. Combining the data from this new analysis, we compose synthesis-structure prediction (SSP) maps, which plot descriptors for the spatial distribution of Zn and Cd as a function of the encoding reaction variables. Finally, we resolve the temporal evolution of the MOF nanoparticle structure using *in situ* XRD measurements, which reveal that the internal architecture is determined by the different crystallisation rates of Zn- and Cd-rich phases, as well as significant intraparticle diffusion of both ions across the core–shell interface. The work provides unprecedented predictive capability for potential nanostructure design and tuning in mixed-component MOFs.

## 2 Results

### 2.1 Phase behaviour and sample inhomogeneity

In order to investigate how reaction conditions affect their phase behaviour, 99 samples of nominal composition Zn_1−*x*_Cd_*x*_(mIm)_2_ were synthesised within a parameter space defined by temperature (20  °C ≤ *T* ≤ 100 °C) and composition (0 ≤ *x*_rxn_ ≤ 1, where *x*_rxn_ = mole fraction of Cd in the reaction mixture). For details see Experimental methods and ESI Section S1.† The reaction duration was chosen to be 24 h—shorter than the reported 48 h syntheses^32,33^ —the consequence of which, as we will come to show, was access to a kinetic synthesis regime and controllable compositional heterogeneity.

**Fig. 1:**
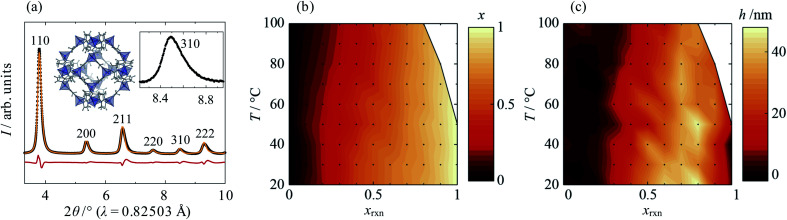
(a) Single-phase split-peak model fit to the high-resolution synchrotron XRD data, (b) average Cd mole fraction, *x*, and (c) peak anisotropy, *h*, as a function of synthesis temperature, *T*, and reaction Cd mole fraction, *x*_rxn_. (a) shows data from synthesis with *x*_rxn_ = 0.5 at 20 °C (*λ* = 0.82503 Å). Experimental, calculated and difference data are shown in black, orange and red, respectively, and peaks are labelled with their corresponding Miller indices. Insets show the crystal structure of Zn-ZIF-8 (Zn, C, H and N atoms are shown in dark blue, grey, light grey and light blue, respectively) and an enlarged view of the 310 peak, which exhibits typical peak asymmetry. In (b) and (c), the *T-x*_rxn_ conditions explored are shown as points and conditions under which phases other than ZIF-8 formed are shown in white.

Close inspection of high-resolution synchrotron diffraction data revealed unusual peak asymmetry in the mixed-component samples, which suggested significant compositional inhomogeneity that was not present in standard samples or the parent materials. Indeed, two-phase refinements using the Pawley method^35^ indicated the existence of Cd-rich and Zn-rich domains, which manifest as overlapping sets of peaks at low angles and high angles, respectively (see ESI Section S2.1†). Peak asymmetry was investigated using a structure-independent single phase split peak profile model, in which the peak broadening at 2*θ* values above and below the peak intensity maxima are independent of each other (Fig. 1a; ESI Section S2.2†). A standard crystallite size convolution was used to model asymmetric peak broadening; the difference in the resulting “crystallite sizes” above and below the peak intensity maxima gave a measure of peak asymmetry, *h*. Using this model, increasing *x*_rxn_ is found to result in a gradual increase in the average lattice parameter from *ca.* 17 Å (pure Zn(mIm)_2_) to 18 Å (pure Cd(mIm)_2_) at all temperatures, as was found in previous reports.^32,33^ According to Vegard's law, this corresponds to an increase in the average mole fraction of Cd in the sample, *x*, from 0 to 1, shown in Fig. 1b. Increasing *T* also results in small decreases in *x*, which indicates that Cd incorporation becomes disfavoured. This is confirmed by solid-state ^15^N CP MAS NMR data, which show a decreasing population of linkers coordinating to Cd as the synthesis temperature, *T*, rises (see ESI Section S3†). Peak asymmetry, *h*, is found to be highest for Cd mole fractions 0.5 < *x*_rxn_ < 0.9 at room temperature and, in general, it decreases with increasing *T* (Fig. 1c).

Also of note was the fact that reactions at elevated *T* with high Cd content—above 50 °C for *x*_rxn_ = 1 and above 80 °C for *x*_rxn_ = 0.9—formed crystalline phases other than Cd-ZIF-8, which again suggests instability of Cd-rich ZIF-8. However, just 10–20% doping of Zn into the Cd-rich system was found to be enough to stabilise the ZIF-8 structure at all temperatures investigated.

The behaviours of *x* and *h* make intuitive sense: at intermediate values of *x*_rxn_ there is the most compositional flexibility to form significant amounts of both Cd- and Zn-rich phases, whilst higher *T* increases homogeneity by improving mixing during crystallisation. However, based on XRD alone, the physical manifestation of phase separation in the samples remained unclear. Therefore, it was necessary to investigate the metal ion distribution on the length scale of individual particles.

### 2.2 Nanoscale core–shell distribution

**Fig. 2:**
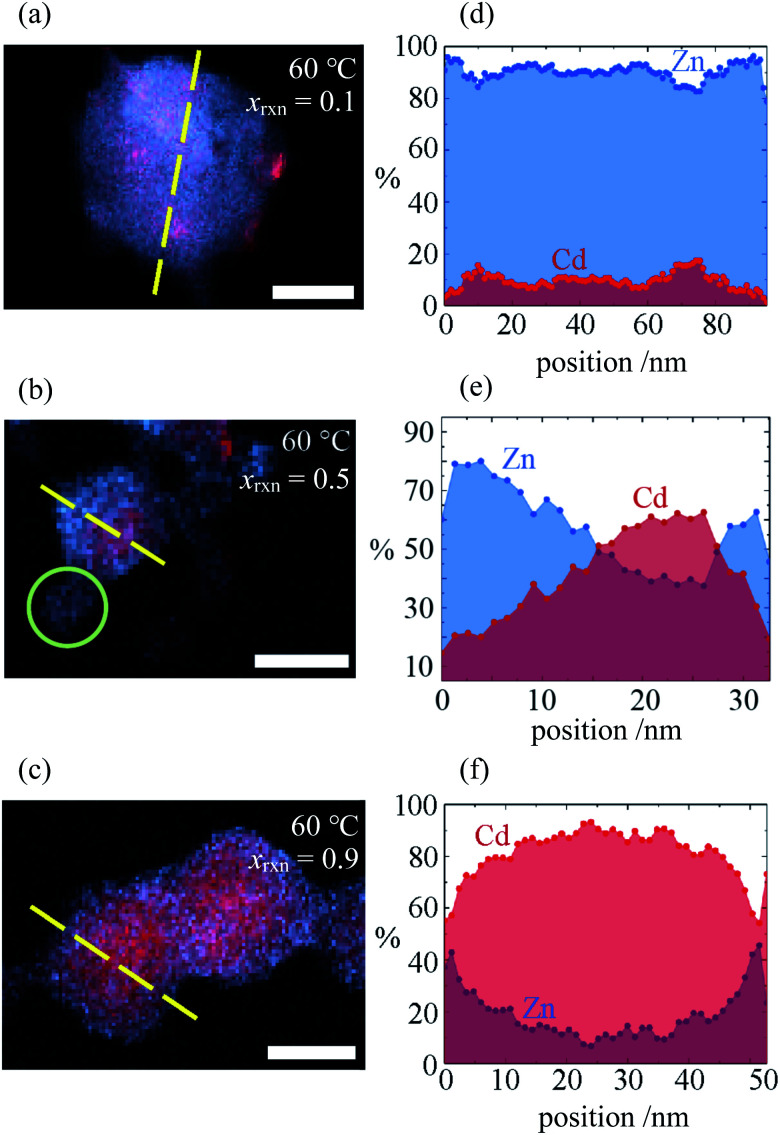
(a–c) STEMEDX images of Zn/Cd ZIF-8 nanoparticles synthesised at *T* = 60 °C with *x*_rxn_ = 0.1, 0.5, and 0.9, respectively, and (d–f) line profiles corresponding to dashed lines in (a–c) showing the percentage of Zn and Cd at each pixel. Scale bars = 30 nm; green circle in (b) indicates possible secondary nucleation of a Zn-rich particle.

Investigation of selected reaction products using STEMEDS showed that the compositional inhomogeneity suggested by the asymmetric XRD peak profiles manifests as nanoparticles with a Cd-rich core, surrounded by a Zn-rich shell (see Fig. 2 and ESI Section S4†). Line profiles through the particles reveal a general preference for Cd in the centre and Zn at the edges, with an interface of variable gradient between them. Whilst a small number of more Zn-rich particles indicates a dispersion of particle compositions, possibly arising from secondary nucleation, the internal structure of the core–shell nanoparticles overall varies systematically with *x*_rxn_ and *T*. Samples synthesised with *x*_rxn_ = 0.1 consist of Zn-rich material surrounding Cd-rich domains of diameter 10–20 nm, which appear as larger aggregates (Fig. 2a). As *x*_rxn_ increases to 0.5 (Fig. 2b) and 0.9 (Fig. 2c), individual core–shell particles become more distinct, and the Cd-rich domains increase in size and Cd content. The core–shell interface appears to be most diffuse for *x*_rxn_ = 0.5. Variations in structure as a function of *T* are relatively subtle (see ESI Fig. S15–S19†). In this case, the overall Cd content appears to decrease with increasing *T*, which is consistent with decreasing values of *x* observed in the XRD data. In addition, increasing *T* results in more interface diffuseness and increased similarity between the core and shell compositions, which would be consistent with decreasing peak asymmetry observed in the XRD data. Particle size distributions are largely consistent with previous observations:^33^ particles increase in size as Cd content increases, whilst size remains constant as *T* increases (ESI Section S4.3†).

The consistent trends in phase behaviour seen in the STEMEDS and XRD data lead to our first key result: that the short, single-step synthesis results in Zn/Cd ZIF-8 nanoparticles with a core–shell structure, which is the probable cause of XRD peak asymmetry. Variations in composition and core–shell structure arise from changes in *x*_rxn_ and *T*. However, owing to the time-consuming nature of STEM–EDS, the statistical investigation of multiple particles and samples was beyond the scope of this work. Therefore, we were motivated to extract structural information directly from XRD peak profiles.

### 2.3 Composition gradient model

**Fig. 3:**
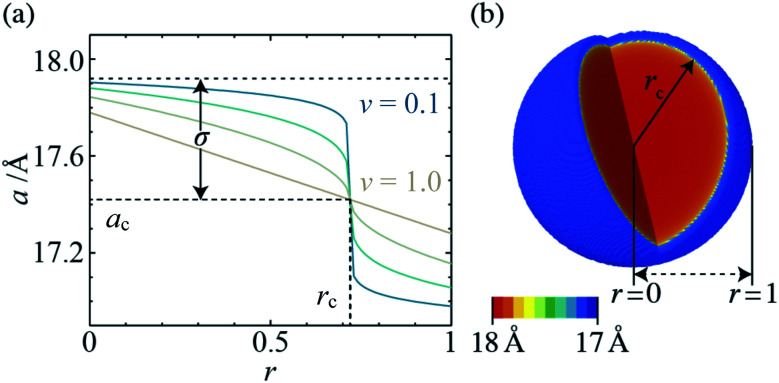
(a) Radial lattice parameter profiles derived using the composition gradient model for *ν* = 0.1, 0.25, 0.5 and 1.0 (*a*_c_ = 17.42 Å, *r*_c_ = 0.72, and *σ* = 0.5), and (b) 3-D lattice parameter distribution, which corresponds to *in situ* XRD data for *x*_rxn_ = 0.5, *T* = 25 °C, *t* = 100 s (*a*_c_ = 17.47 Å, *r*_c_ = 0.94, *σ* = 0.52, and *ν* = 0.13).

**Fig. 4:**
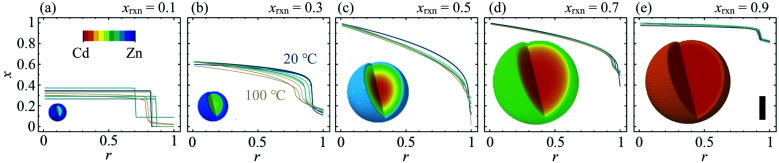
Radial composition profiles determined by composition gradient model fitting, for *x*_rxn_ = 0.1, 0.3, 0.5, 0.7 and 0.9 [(a–e), respectively] at *T* = 20–100 °C. Insets show 3-D composition distributions corresponding to *T* = 60 °C, sizes scaled to the coherent scattering length, *D*. Scale bar = 25 nm.

Informed by the STEMEDS evidence, we devised a new, physically meaningful model to fit the asymmetric XRD peak profiles, which facilitated the extraction of detailed structural information about the diffuse core–shell MOF nanoparticles from the bulk samples. Our composition gradient model relies on the relatively large (6%) difference in lattice parameter between the pure Zn and pure Cd ZIF-8 parent materials and assumes that this is the major contributor to peak asymmetry over any other effects, such as strain in the particles due to lattice mismatch. Samples are modelled as spherical core–shell particles, whose composition—and, therefore, lattice parameter—varies monotonically from the centre (*r* = 0) to the surface (*r* = 1), according to the equation1
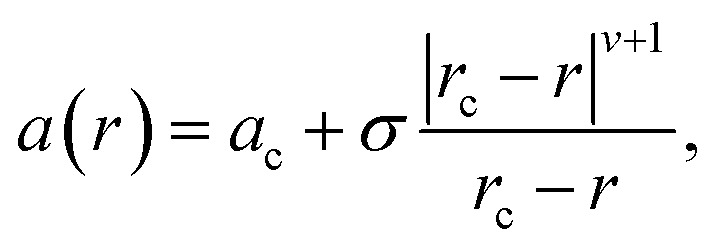
where *a*(*r*) is the lattice parameter at position *r*, and *r*_c_ is the nominal position of the core–shell interface, at which *a*(*r*_c_) = *a*_c_. *σ* describes the function amplitude and *ν* describes its curvature; a sharp interface is represented by a value of *ν* = 0, whilst *ν* = 1 indicates a linear change in *a* from core to shell (see Fig. 3). In order to fit the model to the XRD data, we assign individual crystallographic “phases” to an arbitrary number of discrete shells with outer radius *r*. The lattice parameter and volume contribution of each phase are specified by the values of *r*_c_, *a*_c_, *σ*, and *ν*. These values are refined to fit the XRD data, along with a coherent scattering domain size, *D*, which accounts for particle size broadening, thus defining the radial lattice parameter profile (Fig. 3a) and 3-D distribution (Fig. 3b). Vegard's law can then be used to directly calculate the radial composition profiles and 3-D composition distributions, as shown in Fig. 4. This model was chosen because (i) it uses relatively few parameters to describe the XRD peak profiles (note that the “phases” are highly constrained by eqn (1) and thus increasing their number does not lead to additional fit parameters), and (ii) it accounts for the key scenarios observed by STEM-EDS, *i.e.* inhomogeneity, core–shell structure, and monotonic variation in composition. In principle, it should be applicable to any nanoparticles with such features, providing the space groups of the parent phases are the same and the lattice parameters show measurable variation.

### 2.4 Radial composition profiles

**Fig. 5:**
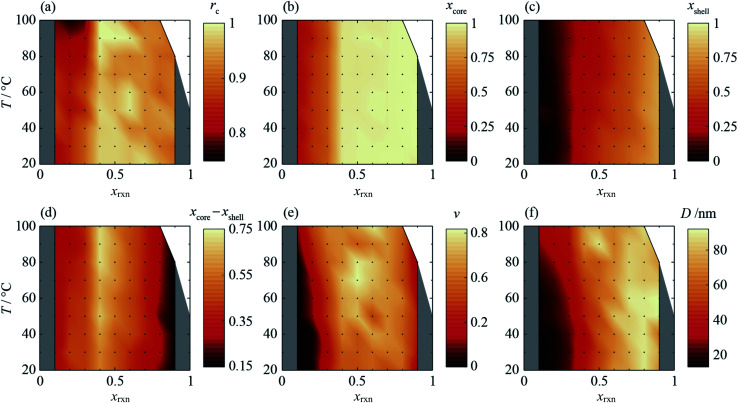
Synthesis-structure prediction (SSP) maps for selected structural characteristics of core–shell Zn/Cd ZIF-8 nanoparticles as a function of synthesis temperature, *T*, and reaction mixture Cd mole fraction, *x*_rxn_: (a) nominal interface radius, *r*_c_, (b) representative core Cd mole fraction, *x*_core_, calculated from radial composition profiles at *r* = 0.05, (c) representative shell Cd mole fraction, *x*_shell_, calculated at *r* = 0.95, (d) core–shell composition difference, *x*_core_ − *x*_shell_, (e) interface diffuseness, *ν*, and (f) coherent scattering length, *D*.

The composition gradient model described above was fit to the high resolution XRD data as outlined in the Experimental methods section, resulting in a radial composition profile and 3-D composition distribution for each particular synthesis condition in *T*–*x*_rxn_ parameter space (see Fig. 4 and ESI Section S5†). The relationships between *T* and *x*_rxn_, and composition, interface radius, diffuseness and particle size were plotted as synthesis–structure prediction (SSP) maps (Fig. 5), thus revealing our second key result: that control over the synthesis conditions affords a direct route to tuning the core–shell interface.

In all samples, the values of *x* found in the core and shell increase as *x*_rxn_ increases and fall either side of the reaction Cd mole fraction, *x*_rxn_ (Fig. 5a and b), mirroring the compositions determined using two-phase refinements (see ESI Section S6†). Whereas the composition range for individual samples with intermediate composition (*e.g.*, *x*_rxn_ = 0.5, see Fig. 4c) spans almost the entire range of possible *x* values, the corresponding range is much reduced in samples with compositions closer to the parent compounds, reflecting trends in the STEMEDS observations. This is apparent in the maximum value of (*x*_core_ − *x*_shell_), which is found at *x*_rxn_ = 0.4, largely independent of *T* (Fig. 5c).

Likewise, the core–shell interface is sharpest in samples with values of *x*_rxn_ close to the parent materials, in which *x*_core_ is largely independent of *r* (Fig. 4a and e). In contrast, intermediate values of *x*_rxn_ lead to more diffuse core–shell interfaces and, within the core, *x* depends strongly on *r* (Fig. 4b–d). Values of *r*_c_ (Fig. 5d) and *ν* (Fig. 5e) are indicative of this diffuseness and are typically largest at intermediate values of *x*_rxn_. Subtle dependences of *r*_c_ and *ν* on *T* are also apparent in samples with low Cd content. For example, when *x*_rxn_ ≤ 0.3, *r*_c_ decreases and *ν* increases as *T* increases, which indicates that the nominal core–shell boundary gets closer to the particle centre and the composition gradient becomes smoother. Finally, it is worth noting that the coherent scattering length, *D*, increases with increasing *x*_rxn_ and, to a lesser extent, with *T* (Fig. 5f). This reflects overall trends in the size of particles observed by STEMEDS and is consistent with previous reports^32,33^ and our own two-phase refinements (see ESI Section S6†), noting that XRD data are largely insensitive to aggregation of particles, as seen in the *x*_rxn_ = 0.1 sample.

### 2.5 Formation kinetics

**Fig. 6:**
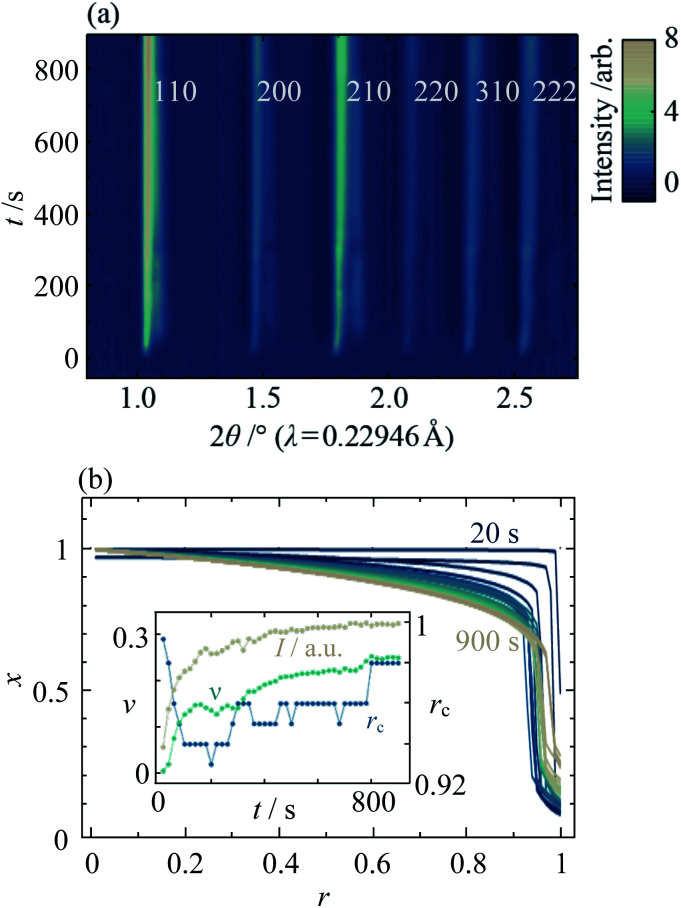
(a) *In situ* XRD data of mixed Zn/Cd ZIF-8 crystallisation as a function of reaction time, *t*, for *x*_rxn_ = 0.5 at *T* = 25 °C, and (b) the corresponding radial composition profiles. Inset shows the evolution of total diffraction peak intensity, *I* (beige), interface diffuseness, *ν* (turquoise), and nominal interface radius, *r*_c_ (blue).

In order to understand the origins of the core–shell MOF nanoparticles, *in situ* synchrotron XRD was used to monitor their crystallisation, finding that the phase separation is essentially kinetic in origin. Data from the mixed-metal reaction of *x*_rxn_ = 0.5 at 25 °C are shown in Fig. 6a and ESI Fig. S32.† One set of peaks at low 2*θ* appears first, followed by a second set of distinct peaks at higher 2*θ* after several seconds. As the reaction continues, the peaks merge gradually to form a single set of peaks with profiles that are somewhat similar to those observed in the *ex situ* patterns. These observations indicate that nucleation and initial growth are dominated by Cd-rich material (larger lattice parameter, peaks at lower 2*θ*). Zn-rich ZIF-8 (peaks at higher 2*θ*) grows more slowly and, given the STEM–EDS results, most probably on the outer surface of existing particles. Material with intermediate composition forms as the reaction proceeds further. At higher temperatures, the growth of peaks is faster (see ESI Section S8†); above 45 °C, the initial growth of the low 2*θ* peaks is too fast to be observed and peak overlap already occurs at the onset of data collection (20 s after mixing). Quantitative analysis of *in situ* data from the parent materials indicates that the rate of formation of Zn-ZIF-8 increases to become competitive with Cd-ZIF-8 at higher temperatures; see ESI Section S9† for further discussion.

Fitting the composition gradient model to the *in situ* XRD data reveals not only that the particles' internal structure evolves *via* a Cd-rich core that becomes surrounded by a Zn-rich shell, but also that significant redistribution of Zn and Cd occurs across the core–shell interface after crystallisation has occurred (Fig. 6b; ESI Section S8†). Initially, *r*_c_ and *ν* take values close to 1 and 0, respectively, indicating that the first crystalline particles to form are pure Cd-ZIF-8 (Fig. 6b, inset). Within tens of seconds, a Zn-rich shell with a well-defined core–shell interface develops, as evidenced by a rapid decrease in *r*_c_ and low values of *ν* ≈ 0.14. At higher temperatures, faster nucleation results in the formation of both core and shell phases before the first data are collected (see ESI Section S8†). Over the next few hundred seconds, the amount of crystalline material continues to increase and the core–shell interface becomes more diffuse, as indicated by increasing values of the total diffraction peak intensity, *I*, and *ν*. Between around 400 s and 800 s, the value of *I* stabilises but *r*_c_ and *ν* continue to rise, indicating that the core–shell interface continues to become more diffuse despite the addition of no more crystalline material. Comparing the final *in situ* radial composition profile with the *ex situ* data in Fig. 4a, it is clear that even after 900 s the metal distribution is not yet stabilised, which indicates that intraparticle diffusion across the core–shell interface continues to occur well into the synthesis.

## 3 Discussion

The above *in*- and *ex situ* XRD data demonstrate that the spatial distribution of Zn and Cd in mixed-component ZIF-8 nanoparticles can be finely tuned *via* the reaction conditions (*x*_rxn_, *T* and *t*) of a single-step synthesis. In the context of materials design, the arising predictability and control over physical structure is highly desirable as it should lead to rational improvements in, for example, gas sorption and separation performance. The SSP maps also reveal some important limitations of the single-step synthesis strategy, such as the absence of synthesis conditions within the explored *T*–*x*_rxn_ parameter space that produce particles with a Zn-rich core or particles with both a homogeneous core and a large difference between the core and shell compositions. Elucidation of multi-step fabrication routes may be a way to overcome such limitations, albeit at a cost to synthetic simplicity.

The *in situ* measurements provide the important understanding of the microscopic origins of the Zn/Cd ZIF-8 phase separation, required if this new single-step strategy is to be generalised to other mixed-component MOFs. Clearly, both chemical kinetics and thermodynamics are important.^28^ Ostwald's rule of stages^36^ favours faster nucleation of phases with lower stability, in this case Cd-ZIF-8 over Zn-ZIF-8. Considering ligand exchange kinetics, the lower charge density of Cd^2+^ compared to Zn^2+^ promotes faster assembly of Cd(mIm)_*x*_-like pre-nucleation species^37^ and Cd-ZIF-8 nuclei compared to their Zn-based counterparts. Faster diffusion of Cd(mIm)_*x*_-like units, also arising from their lower charge density, and better lattice matching will lead to growth of Cd-rich particles—the origin of the Cd-rich core in the mixed-metal nanoparticles—and depletion of Cd^2+^ in the surrounding volume. The observed increase in particle size as *x*_rxn_ increases may be caused by a faster increase in the rate of crystallite growth of the Cd-rich particles compared to the rate of their nucleation. Similar trends in particle size have been observed for Zn/Co-^38^ and Zn/Cu-ZIF-8 nanoparticles.^39^

At room temperature, the Zn^2+^ species that remain in the depleted solution attach more slowly and to existing particle surfaces to give rise to distinct Zn-rich shells, largely in preference to homogeneous nucleation of new particles. An alternative formation mechanism, in which both Cd-rich and Zn-rich particles form and core–shell particles grow at the expense of the Zn-rich particles *via* Ostwald ripening, is unlikely to lead to such a prevalance of core–shell particles with evenly distributed Zn-rich shells as found by STEMEDS. However, this mechanism cannot be entirely ruled out. Indeed, the processes that underpin the formation of Zn-rich ZIF-8 become faster with increasing temperature, thereby increasing the prevalence of more diffuse core–shell interfaces and secondary Zn-rich nuclei. Once the particles are formed, lability of metal–imidazolate bonds^40^ and mass transport on both sides of the core–shell interface lead to continued change towards the thermodynamic ground state, which is a near-homogeneous distribution of ions.^33^ At higher temperatures, mixing is enhanced by the increasing *T*Δ*S* contribution to the free energy and faster metal–ligand exchange, leading to more diffuse interfaces and greater homogeneity. This step is likely to have some mechanistic similarities with “post-synthetic” metal exchange observed previously in ZIFs^41^ and may be arrested upon removal of the MOF from the reaction mixture.

## 4 Conclusions

In summary, we have demonstrated for the first time the synthesis of mixed-component core–shell MOF nanoparticles with a tunable internal interface using a single-step kinetic regime. The composition and interface structure of the particles, which adopt the ZIF-8 crystal structure and comprise a Cd-rich core surrounded by a Zn-rich shell, can be rationally controlled by temperature, time and reactant ratio. We observed this unusual compositional heterogeneity using STEMEDS, and applied a new compositional gradient model to fit to high resolution XRD data for multiple bulk samples. Thus, we were able to reveal the important trends in core–shell features across synthetic parameter space spanning temperature (20 °C ≤ *T* ≤ 100 °C) and composition (0 ≤ *x*_rxn_ ≤ 1), which were illustrated in SSP maps, thus providing predictive capability for potential nanostructure design.

**Fig. 7:**
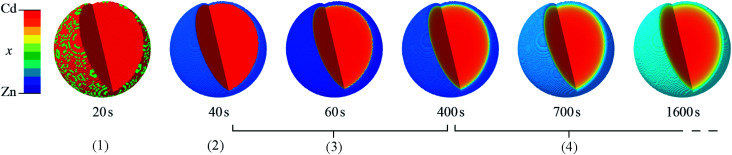
Evolution of core–shell Zn/Cd ZIF-8 particle structure, derived by composition gradient model fits to time-resolved *in situ* synchrotron XRD data for *x*_rxn_ = 0.5 at *T* = 25 °C. Note that particles are not scaled according to size. Stages (1)–(4) represent the nucleation of the Cd-rich core, growth of the Zn-rich shell, increasing interface diffuseness accompanying continued particle growth, and increasing interface diffuseness beyond the end of crystallisation, respectively.

Regarding structure control, the five main findings are as follows: firstly, particles with a distinct core–shell distribution are generated by all values of *x*_rxn_ > 0.1 and both the Cd-rich core and Zn-rich shell increase in Cd content as *x*_rxn_ increases. At *x*_rxn_ = 0.1, small Cd-rich domains appear to be aggregated in larger particles surrounded by Zn-rich material. Secondly, the difference between core and shell compositions is largest for intermediate values of *x*_rxn_ but manifests with a more diffuse interface compared to more extreme values of *x*_rxn_. Thirdly, as *T* increases, the core–shell interface moves towards the centre of the particles and becomes more diffuse, owing to the greater kinetic and thermodynamic driving forces for mixing. Fourthly, as *x*_rxn_ increases, the coherent scattering length, *D*, increases, in agreement with our initial XRD and STEMEDS analyses and previous reports of increasing particle size.^32,33^ Finally, *in situ* XRD showed that the spatial distribution of metals within the nanoparticles arises from the rapid formation of a Cd-rich nucleus followed by growth of the Zn-rich shell. Then as the reaction continues, the lability of the MOF structure allows significant diffusion to occur across the interface as the spatial distribution of metals tends towards the well-mixed thermodynamic state. This evolution is shown in Fig. 7.

We believe this to be the first conclusive evidence of nanoscale core–shell partitioning in mixed-component MOFs realised in a single synthetic step, which offers important advantages of reduced complexity and simpler processing compared to multi-step fabrication.^42^ Whilst previous reports of core–shell MOF particles rely on highly differential crystallisation kinetics between components,^7–9^ it is clear that the synthesis conditions used herein support a crystallisation regime that is highly susceptible to perturbation and, importantly, this is key to the precise control over interface structure. Furthermore, by quenching the formation process before it reaches equilibrium, we have achieved kinetic partitioning of Cd- and Zn-rich domains on a much finer 10–50 nm lengthscale. We envisage that even finer—or indeed, coarser—control over heterogeneity may be achievable in a single step for a range of systems, by selecting reaction parameters that lead to equally perturbable kinetic synthesis regimes.

Possible advantages of a radial composition gradient include the potential to continuously vary lattice parameters in one particle,^43^ which will improve tuning of adsorption kinetics for separations,^7^ or size-selectrive catalysis.^9,26^ Other properties, including electronic conductivity, electrocatalytic activity and thermal, chemical or mechanical stability,^44^ may be dominated by the interface and, thus, also be optimised in this way for potential applications, such as artificial photosynthesis. In addition, this work demonstrates the possibility of tuning and structure control beyond existing methods, which may result in a new generation of nanostructured MOFs with improved performance and novel behaviours for as-yet unimagined applications.

Finally, we note that the details revealed by applying our new composition gradient model to XRD data are rarely achievable using other bulk techniques save, for example, high resolution small angle scattering. In future, the potential to combine it in the future with crystallographic insight *via*, for example, Rietveld refinement to the same XRD dataset, should enable even finer control over mixed-component MOF structures.

## 5 Experimental methods

Samples were prepared using a modification of a previously reported solvothermal route;^33^ full details of all experimental methods are given in ESI Section S1.† Briefly, 0.1 M Zn(NO_3_)_2_·6H_2_O (Sigma Aldrich, ≥99%) and 0.1 M Cd(NO_3_)_2_·4H_2_O (Sigma Aldrich, 98%) were combined to give a total volume of 5 cm^3^, and combined with 5 cm^3^ of 0.8 M 2-methylimidazole (Sigma Aldrich, 99%) and 0.8 M triethylamine (Alfa Aesar, 99%). Reactions were performed at temperatures between 20 °C and 100 °C for 24 h. Phase space was mapped in steps of 10 °C and *x*_0_ = 0.1, respectively. Solid products were separated by centrifugation, washed three times with methanol and dried *in vacuo* overnight.

High resolution XRD patterns were obtained from ground samples packed into 0.5 mm capillaries using the Mythen-2 PSD at the I11 beamline at the Diamond Light Source, UK,^45,46^ with wavelengths of *ca.* 0.825 Å. *In situ* XRD data were collected on beamline I12,^47^ for reactions scaled down to 3 cm^3^ volume at temperatures between 25 °C and 65 °C in 10 °C intervals. A Pilatus 2 M CdTe detector was used with X-rays of wavelength *λ* = 0.22946 Å to produce raw 2-D diffraction data, which were processed in 20 s bins using DAWN.^48–50^

Pawley refinements^35^ were implemented in TOPAS Academic v6.0.^51^ Single and two-phase refinements were carried out using the symmetric Thompson-Cox-Hastings-Pseudo-Voigt (TCH-PV) peak shape,^52^ and split-peak refinements were implemented using the split Pseudo-Voigt peak shape. Composition gradient refinements were carried out using 50 constrained “phases” corresponding to particle shells with radii from *r* = 0 to *r* = 1 and a constant spacing *δ*r = 0.02. The relative intensity contributions of each phase to a given *hkl* peak were determined by multiplying the shell surface area element (∝*r*^2^) by the weighted average of the peak intensities found for the pure Zn and pure Cd samples, *i.e. I*_*hkl*_ ∝ (*I*_*hkl*,Zn_(1 − *x*) + *I*_*hkl*,Cd_(*x*)) × *r*^2^. Line broadening was modelled by convoluting the fixed instrumental broadening, determined using a Si standard, with sample broadening using the “Crystallite” macro in TOPAS^51^ with a single refineable term, *D*, used for all “phases” in a given sample. *In situ* refinements required *D* to be fixed for a given reaction, owing to low signal to noise and larger intrinsic instrument broadening.

STEM data were acquired using an FEI Osiris microscope (Thermo Fisher) equipped with a high-brightness X-FEG electron source operated at 80 kV, using a beam convergence semi-angle of 11.0 mrad. EDS measurements were collected by a Super-X detector system with four detectors symmetrically mounted about the optic axis. The beam current used was approximately 150 pA. The pixel size was between 1 nm and 2 nm and the dwell time per pixel was 120 ms. STEM images were collected before, during, and after EDS acquisition using an annular dark field detector, in order to allow for the correction of the data for sample stage drift. Data were processed using Hyperspy^53^ software, using K_α_(Zn) and L_α_(Cd) X-ray emission lines to generate EDS maps, which were corrected for sample drift using image registration routines in Matlab (Mathworks).

## Conflicts of interest

There are no conflicts to declare.

## Supplementary Material

SC-012-D0SC03940C-s001

SC-012-D0SC03940C-s002
