# Supplementary material for: Single-step synthesis and interface tuning of core–shell metal–organic framework nanoparticles
Source: Chem Sci. 2021 Feb 9;12(12):4494–502. doi: 10.1039/d0sc03940c (PMC8179513; doi:10.1039/d0sc03940c)
Supplement: SC-012-D0SC03940C-s002 [file SC-012-D0SC03940C-s002.pdf]

Electronic Supplementary Material (ESI) for Chemical Science.
This journal is © The Royal Society of Chemistry 2021

Supporting Information

Single-step synthesis and interface tuning of core–shell metal–organic framework nanoparticles

Kieran W. P. Orr,[a,b] Sean M. Collins,[c,d] Emily M. Reynolds,[a,e] Frank Nightingale,[a] Hanna L. B. Boström,[a,f] Simon J. Cassidy,[a] Daniel M. Dawson,[g] Sharon E. Ashbrook,[g] Oxana V. Magdysyuk,[h] Paul A. Midgley,[c] Andrew L. Goodwin,[a] and Hamish H.-M. Yeung*[a,i]

[a] Inorganic Chemistry Laboratory, University of Oxford, South Parks Road, Oxford, OX1 3QR, UK.

[b] Cavendish Laboratory, University of Cambridge, 19 JJ Thomson Avenue, Cambridge, CB3 0HE, UK.

[c] Department of Materials Science and Metallurgy, University of Cambridge, 27 Charles Babbage Road, Cambridge, CB3 0FS, UK.

[d] School of Chemical and Process Engineering & School of Chemistry, University of Leeds, LS2 9JT, UK.

[e] ISIS Neutron and Muon Facility, STFC Rutherford Appleton Laboratory, Chilton, Didcot, Oxon, OX11 0QX, UK.

[f] Max Planck Institute for Solid State Research, Heisenbergstrasse 1, 70569 Stuttgart, Germany.

[g] Department of Chemistry, University of St Andrews, North Haugh, St Andrews, KY16 9ST, UK.

[h] Diamond Light Source Ltd., Harwell Science and Innovation Campus, Didcot, OX11 0DE, UK.

[i] School of Chemistry, University of Birmingham, Haworth Building, Edgbaston, Birmingham, B15 2TT, UK. Tel: 0121 414 8811; email: h.yeung@bham.ac.uk.

Contents

S1 Experimental methods **s3**
S1.1 Synthesis s3
S1.2 X-ray diffraction s3
S1.3 Electron microscopy s4
S1.4 Nuclear magnetic resonance spectroscopy s5

S2 Refinements to high-resolution *ex situ* synchrotron XRD data **s6**
S2.1 Two-phase model s6
S2.2 Single phase split peak model s8
S2.3 Composition gradient model s10

S3 Solid state nuclear magnetic resonance **s12**

S4 Additional STEM–EDS images **s14**
S4.1 STEM–EDS images and line profiles s14
S4.2 Individual ADF images, EDS maps and composite images s18
S4.3 Particle size distributions s22

S5 Radial composition profiles for all samples **s31**

S6 Synthesis–structure prediction maps relating two-phase model refinements **s32**

S7 Composition gradient refinement fits to *in situ* XRD data **s34**

S8 *In situ* XRD data for Zn/Cd ZIF-8 crystallisation above 25 °C **s35**

S9 Kinetic analysis of parent Zn- and Cd-ZIF-8 crystallisation from *in situ* XRD data **s39**

S10 Bibliography **s41**

S1 Experimental methods

S1.1 Synthesis

$Zn_{1-x}Cd_x(mIm)_2$ samples were prepared using a modification of a previously reported solvothermal route.[1] The following solutions were prepared in methanol (Sigma Aldrich, $\geq$ 99 %): (i) 0.1 M $Zn(NO_3)_2{\cdot}6H_2O$ (Sigma Aldrich, $\geq$ 99 %) (ii) 0.1 M $Cd(NO_3)_2{\cdot}4H_2O$ (Sigma Aldrich, 98 %) (iii) 0.8 M 2-methylimidazole (HmIm) (Sigma Aldrich, 99 %), 0.8 M triethylamine (TEA) (Alfa Aesar, 99 %). Solutions containing the Zn and Cd salts were combined with the starting Cd mole fraction, x_{rxn}, varied between 0 and 1 in 0.1 intervals to give a total volume of 5 cm^3 in 20 cm^3 PTFE autoclave liners. 5 cm^3 of the HmIm/TEA solution was added and the reaction mixture was stirred at 500 rpm for two minutes at room temperature. The liners were transferred to autoclaves and heated to a range of temperatures (20 °C–100 °C in 10 °C intervals) in an oven for 24 hr. The heating and cooling rates for all reactions were 200 °C/hr and 80 °C/hr respectively. The resulting solids formed suspensions of varying stability depending on the composition and synthesis conditions; in general the solid sedimented more quickly as Cd content and synthesis temperature increased. These suspensions were centrifuged at 9500 rpm for 10 min, the supernatant discarded and the sediments resuspended in fresh methanol to wash. In total, three cycles of washing were completed before samples were dried overnight in a vacuum oven at room temperature.

S1.2 X-ray diffraction

High resolution *ex situ* XRD patterns were obtained at the I11 beamline at the Diamond Light Source, UK.[2,3] Samples were finely ground and packed into 0.5 mm external diameter borosilicate capillaries (Capillary Tube Supplies Ltd.). Patterns were measured using a position sensitive detector (PSD) made up of Mythen-2 Si μ strip modules. Wavelengths used were 0.82460, 0.82444, 0.82503, and 0.82506 Å, depending on beamtime dates. *In situ* PXRD measurements were taken at the I12 beamline at the Diamond Light Source, UK.[4] Monochromatic X-rays of wavelength $\lambda = 0.22946$ Å were used, and patterns were recorded on a Pilatus 2M CdTe detector. Reactions for $x_{\mathrm{rxn}} = 0.5$, as well as the pure Zn and Cd end-members, were performed at synthesis temperatures between 25 °C and 65 °C in 10 °C steps, whilst high energy synchrotron XRD patterns were collected simultaneously and subsequently summed to give 20 s temporal resolution. Reactions were contained in culture tubes (Duran™) held at constant temperature in an Al heater block under constant stirring, with a thermocouple to measure the solution temperature. All solutions were prepared the same as for *ex situ* reactions. For each reaction, 1.5 cm^3 of the HmIm/TEA solution was injected into 1.5 cm^3 of the metal solution using a syringe pump.

Pawley refinements[5] were implemented in TOPAS Academic (version 6.0).[6] Single phase refinements were carried out initially using a symmetric Thompson-Cox-Hastings-Pseudo-Voigt (TCH-PV) peak shape.[7] Two-phase refinements were performed by fixing the ratio of the intensities for peak hkl to $h'k'l'$ for both phases so the relative intensities of the peaks within each phase were consistent. To a first approximation this holds true because the variation in composition between the two isomorphic phases is small ($\Delta x \approx 0.2$). Split peak refinements were implemented using the split Pseudo-Voigt (`spv`) peak shape, in which peak

profiles are split at the maximum peak intensity and broadening is modelled as a crystallite size effect according to the Scherrer equation. We define h as the difference between the crystallite sizes, *i.e.*, coherent scattering lengths, of the high- and low-angle components. Composition gradient refinements were performed as detailed in the main text, using 50 "phases", evenly spaced at $r = 0.02, 0.04, ..., 0.98, 1$. The scattering factor of each shell is weighted by the volume of its surface area element ($A\delta r \propto r^2$) and squared again to give its intensity contribution to the XRD data. We convolute a fixed contribution from the instrumental line broadening with sample broadening from the "phases" *via* the `Crystallite_Size` macro in Topas Academic V6[6], using a single refinable term, D, according to the expression $lor\_fwhm = 0.1(180/\pi)(\lambda/D\cos\theta)$. D was not refined for *in situ* data because the low signal-to-noise ratio rendered the refinement unstable.

S1.3 Electron microscopy

An FEI Osiris microscope (Thermo Fisher) equipped with a high-brightness X-FEG electron source operated at 80 kV was used to acquire STEM data using a beam convergence semi-angle of 11.0 mrad. EDS measurements were collected by a Super-X detector system with four detectors symmetrically mounted about the optic axis. The beam current used was approximately 150 pA. The pixel size was 1 nm–2 nm and the dwell time per pixel was 120 ms. Under these conditions, the MOF crystals undergo loss of crystal structure and ionization damage. However, minimal migration of metal ions is observed in MOFs under these conditions[8] borne out by systematic differences observed between samples prepared under controlled synthetic parameters. STEM images were collected before, during, and after EDS acquisition using an annular dark field detector, in order to allow for the correction of the data for sample stage drift during STEM–EDS map aquisition.

Data were processed using the Hyperspy[9] open source software. Integration of K_α(Zn) and L_α(Cd) X-ray emission lines generated the EDS maps, which were corrected for sample drift using image registration routines in Matlab (Mathworks). The Cliff-Lorimer 'k-factor' approach[10] was implemented for quantification using constants supplied by Bruker (the detector manufacturer) with background subtraction by linear interpolation between adjacent energy windows applied to the selected X-ray lines. Due to errors in k-factors, especially for the L_α emission lines, it is known that 10 % uncertainty or greater may be expected in quantification results.[11] However, the precision recorded in changes between a systematic series of similar samples is instead limited by counting statistics due to the particle size, electron beam current, and detector collection efficiency. This precision is estimated from the local fluctuations in composition as approximately 5 %. The maps and line profiles, which were extracted along paths traversing single particles identified from annular dark field STEM imaging, therefore reliably track changes in the projected core–shell composition under different synthesis conditions. It should be noted that the two-dimensional maps can be considered as projections of the three-dimensional composition along the electron beam trajectories (*i.e.*, the map pixels). Therefore, core compositions are underestimated relative to the physical three-dimensional composition.

Particle size analysis was carried out using ImageJ software. One measurement of particle 'diameter' was taken for each discernible primary particle as a line from edge to edge across the centre of the projection of the particle in the ADF–STEM image. Care was taken to

ensure this measurement was an intermediate estimate between short and long dimensions, a suitable choice to capture trends in size distributions given the particles were characterised by convex and compact features with minor deviations in aspect ratio.

S1.4 Nuclear magnetic resonance spectroscopy

Solid-state NMR spectra were recorded using a Bruker Avance III spectrometer equipped with a 9.4 T wide-bore superconducting magnet, giving Larmor frequencies of 400.1, 100.6 and 40.5 MHz, respectively, for ^{1}H, ^{13}C and ^{15}N. Samples were packed into standard 4 mm zirconia rotors and rotated at magic angle spinning (MAS) rates of 5 kHz (^{15}N) or 12.5 kHz (^{13}C) using a Bruker "low-γ" double-resonance probe. ^{13}C NMR spectra were recorded with cross polarisation (CP) from ^{1}H, with a contact pulse (ramped for ^{1}H) of 2.5 ms and signal averaging for 256–1024 transients with a recycle interval of 3 s. ^{15}N CP MAS NMR spectra were recorded with a contact pulse (ramped for ^{1}H) of 5 ms and signal averaging for 1024–22528 transients with a recycle interval of 3 s. High-power TPPM-15 decoupling of ^{1}H was carried out during acquisition. Chemical shifts are reported relative to $(CH_3)_4Si$ using L-alanine as a secondary reference ($\delta(\mathbf{C}H_3) = 20.5$ ppm) for ^{13}C and to CH_3NO_2, using ^{15}N-enriched glycine as a secondary reference ($\delta(\mathbf{N}H_3) = -347.4$ ppm) for ^{15}N.

S2 Refinements to high-resolution *ex situ* synchrotron XRD data

Representative fits to the XRD data obtained using the two-phase, single phase split peak, and composition gradient models, are shown below for samples synthesised at T = 20 °C, 60 °C, and 100 °C; x_{rxn} = 0.1, 0.5 and 0.9.

S2.1 Two-phase model

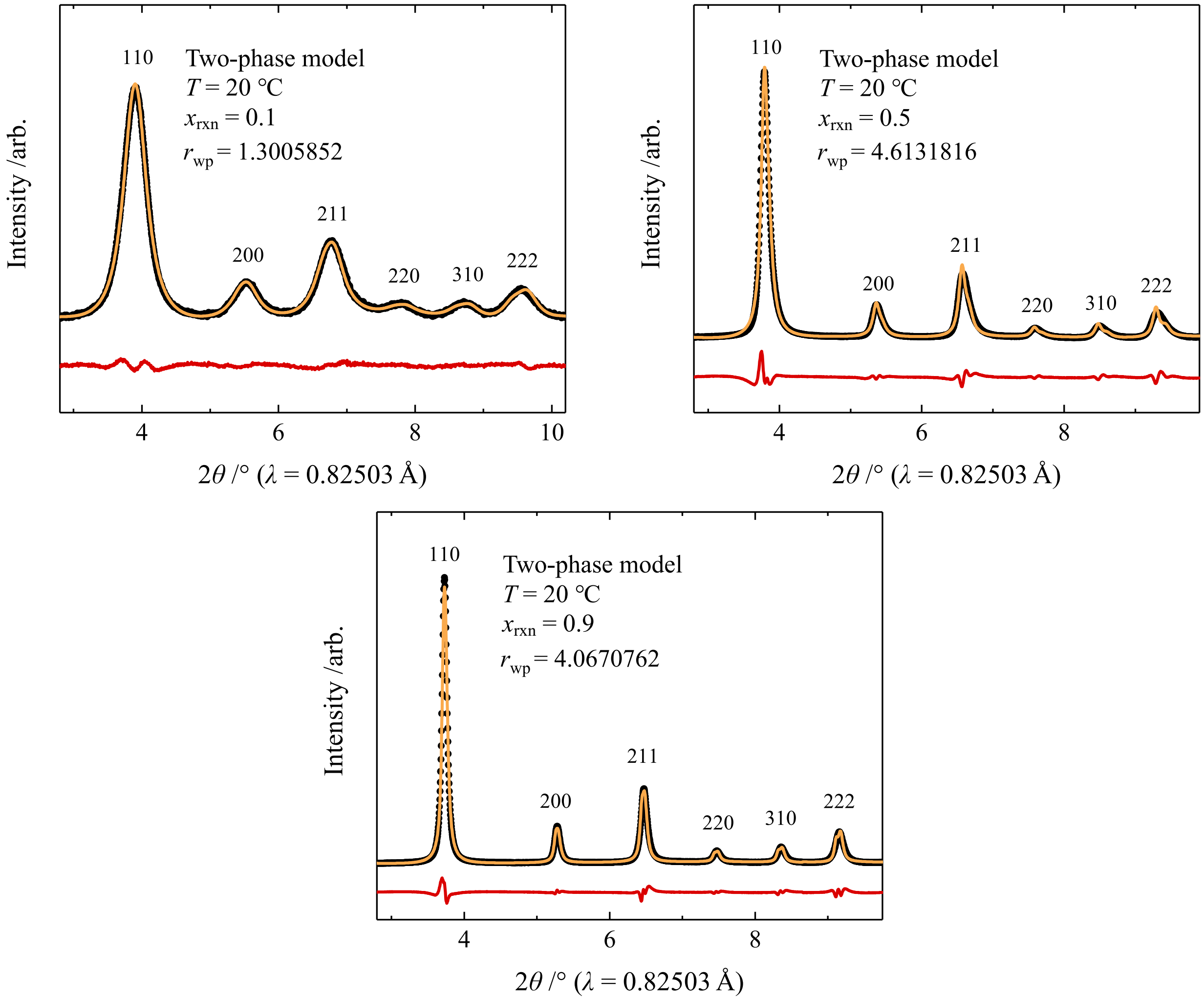

Figure S1: Two-phase fits to high-resolution XRD data for samples synthesised at T = 20 °C; x_{rxn} = 0.1, 0.5 and 0.9. Observed, calculated and difference data are shown as black dots, and orange and red lines, respectively.

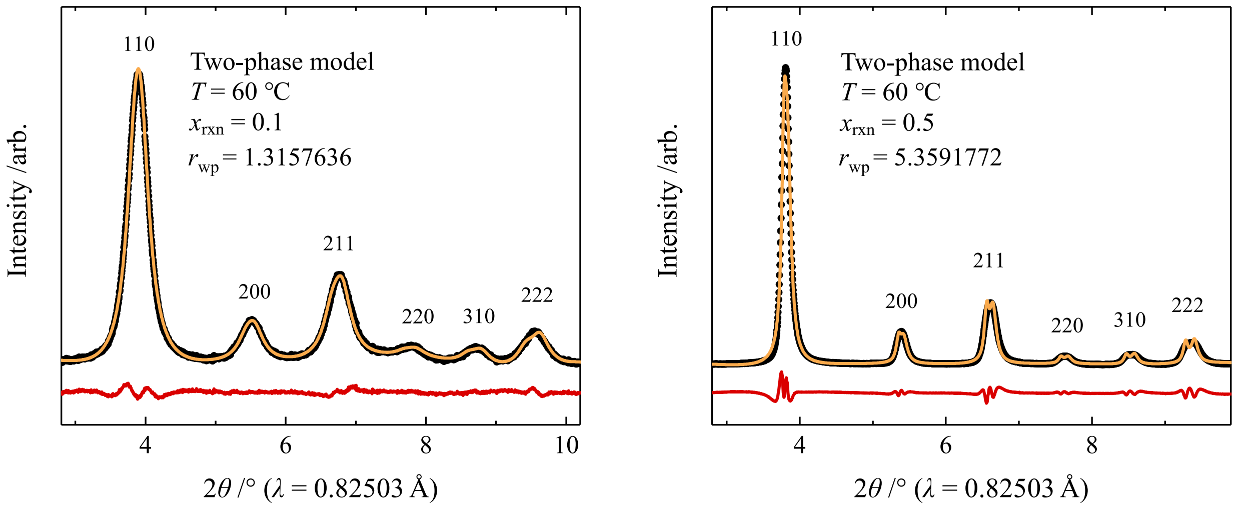

Figure S2: Two-phase fits to high-resolution XRD data for samples synthesised at $T = 60\,°C$; $x_{rxn} = 0.1$ and 0.5. Observed, calculated and difference data are shown as black dots, and orange and red lines, respectively.

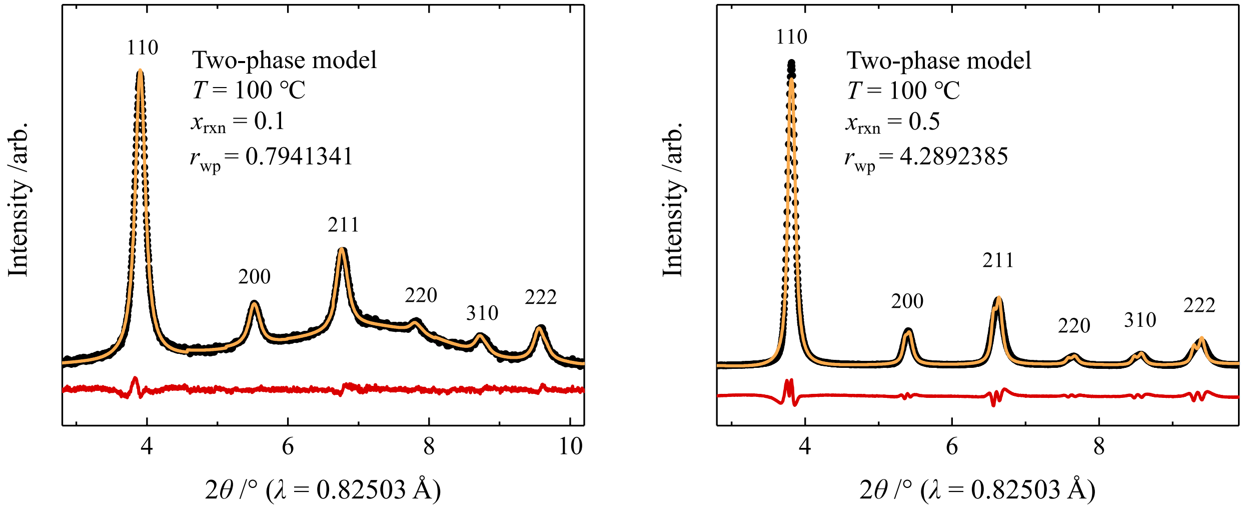

Figure S3: Two-phase fits to high-resolution XRD data for samples synthesised at $T = 100\,°C$; $x_{rxn} = 0.1$ and 0.5. Observed, calculated and difference data are shown as black dots, and orange and red lines, respectively.

S2.2 Single phase split peak model

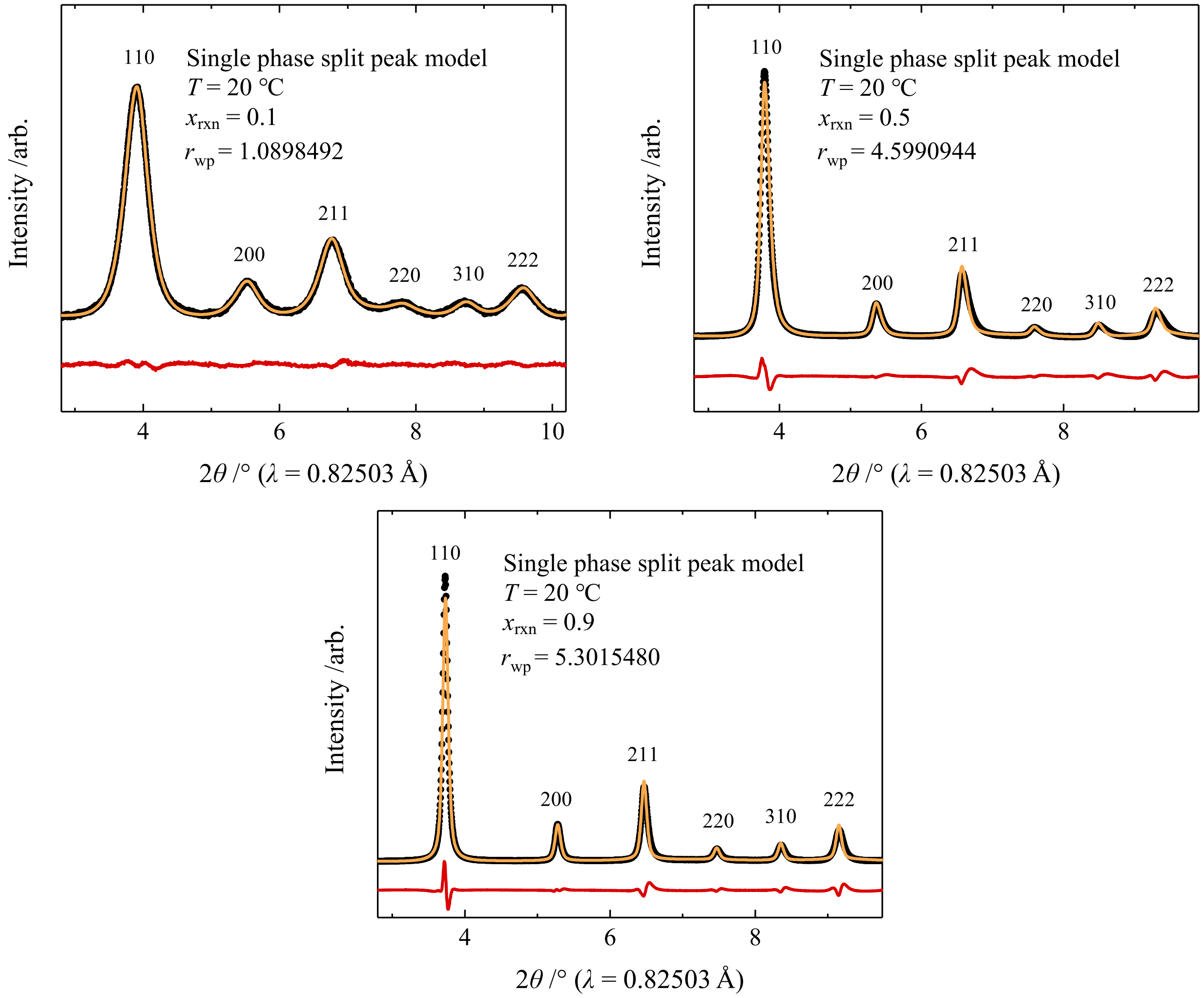

Figure S4: Single phase split peak fits to high-resolution XRD data for samples synthesised at $T = 20\,^{\circ}\mathrm{C}$; $x_{rxn} = 0.1$, 0.5 and 0.9. Observed, calculated and difference data are shown as black dots, and orange and red lines, respectively.

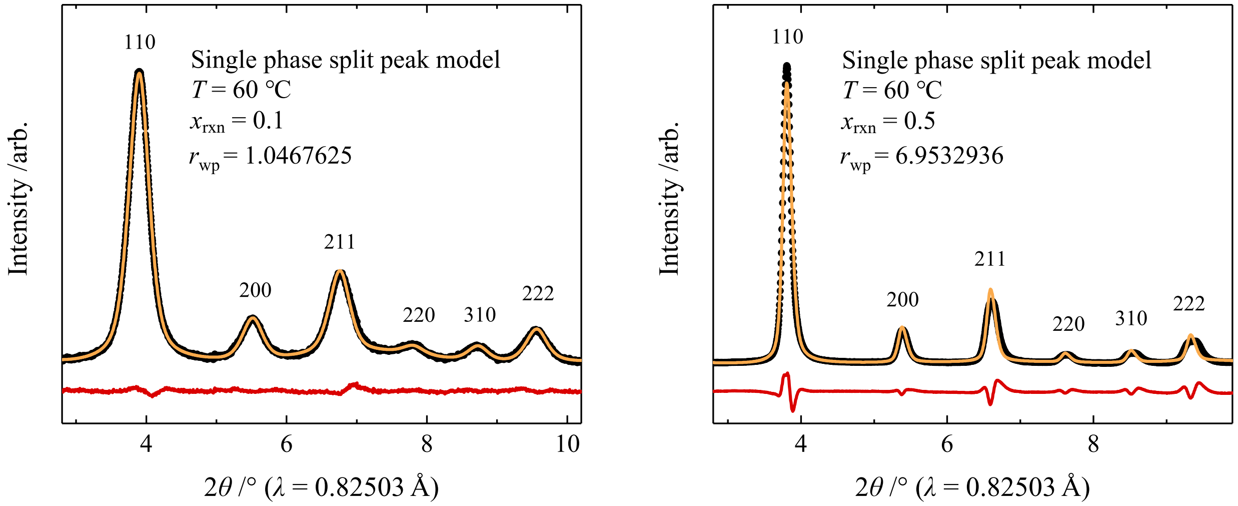

Figure S5: Single phase split peak fits to high-resolution XRD data for samples synthesised at $T = 60\,^{\circ}\mathrm{C}$; $x_{\mathrm{rxn}} = 0.1$ and 0.5. Observed, calculated and difference data are shown as black dots, and orange and red lines, respectively.

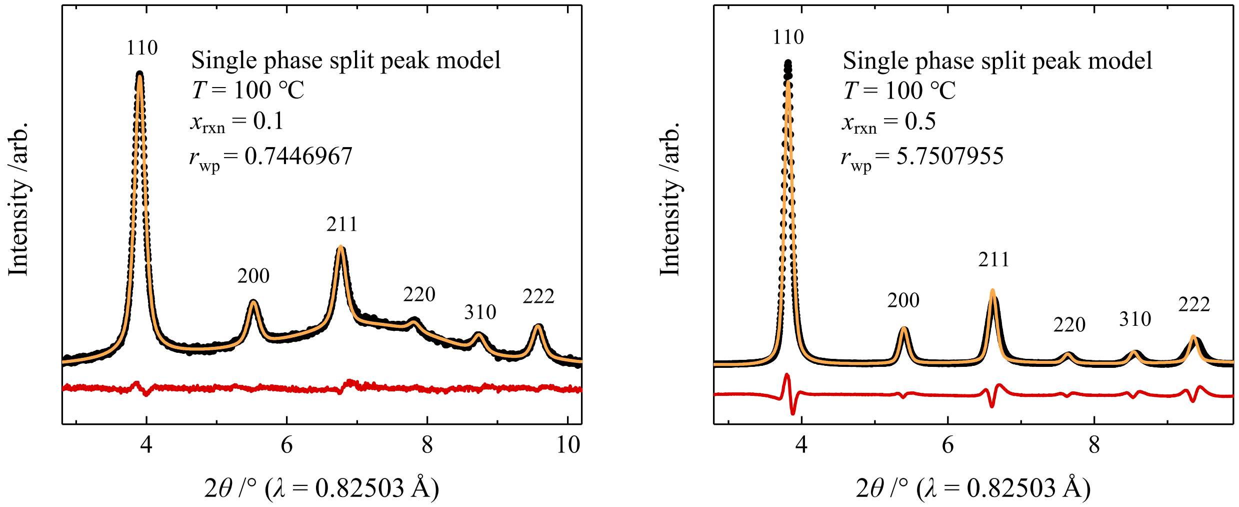

Figure S6: Single phase split peak fits to high-resolution XRD data for samples synthesised at $T = 100\,^{\circ}\mathrm{C}$; $x_{\mathrm{rxn}} = 0.1$ and 0.5. Observed, calculated and difference data are shown as black dots, and orange and red lines, respectively.

S2.3 Composition gradient model

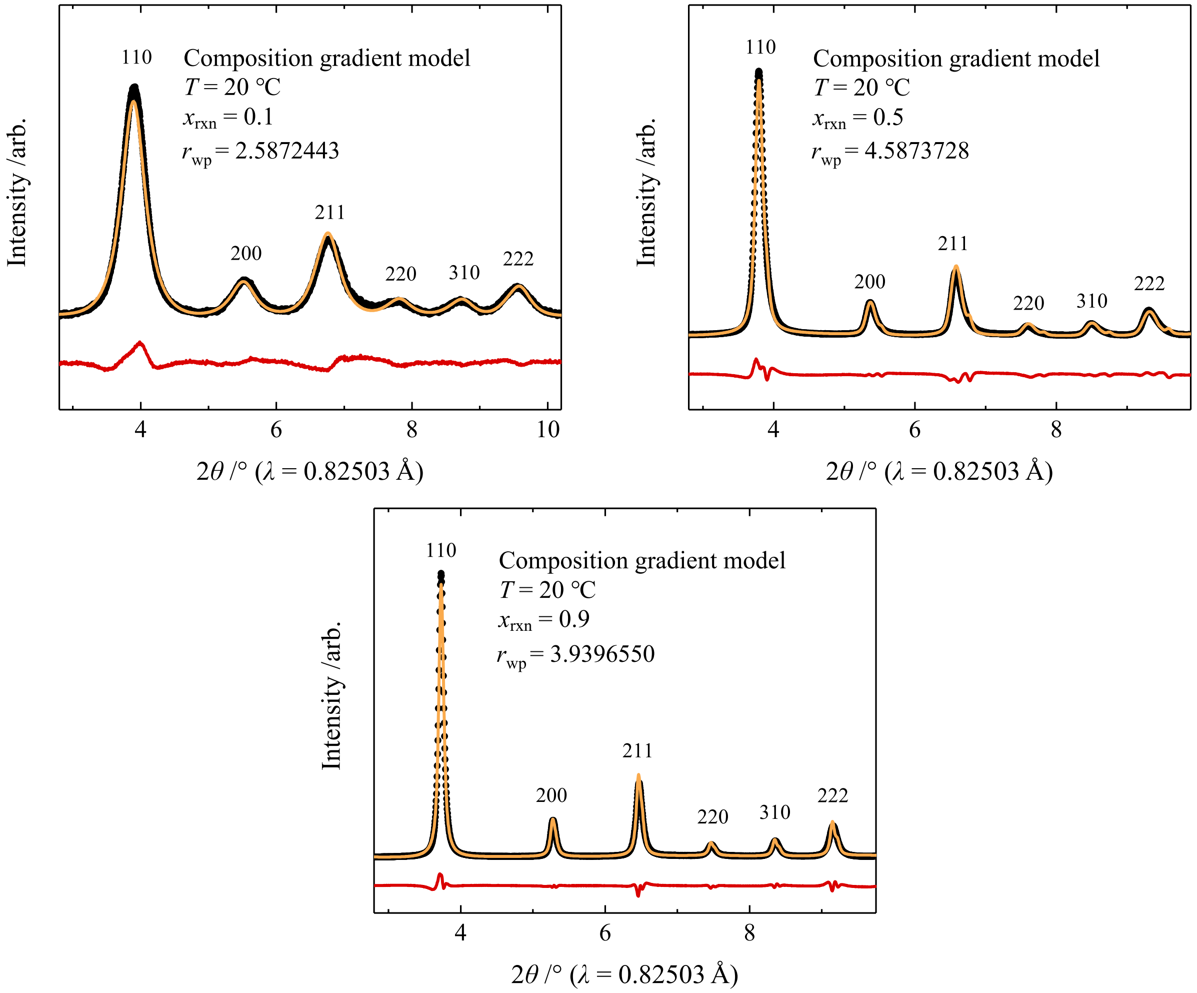

Figure S7: Composition gradient fits to high-resolution XRD data for samples synthesised at $T = 20\,°C$; $x_{rxn} = 0.1$, 0.5 and 0.9. Observed, calculated and difference data are shown as black dots, and orange and red lines, respectively.

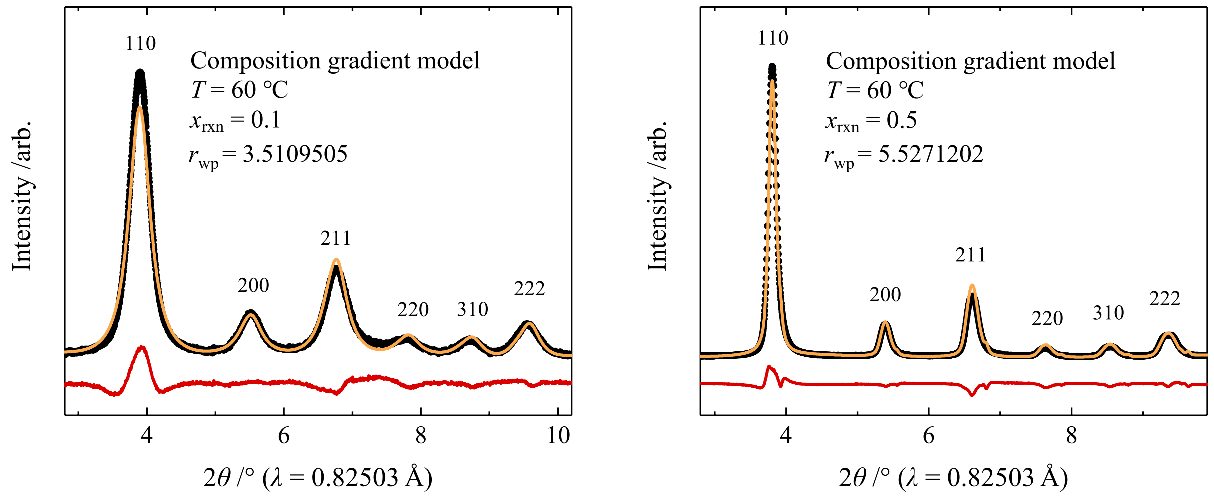

Figure S8: Composition gradient fits to high-resolution XRD data for samples synthesised at $T = 60\,°\mathrm{C}$; $x_{\mathrm{rxn}} = 0.1$ and 0.5. Observed, calculated and difference data are shown as black dots, and orange and red lines, respectively.

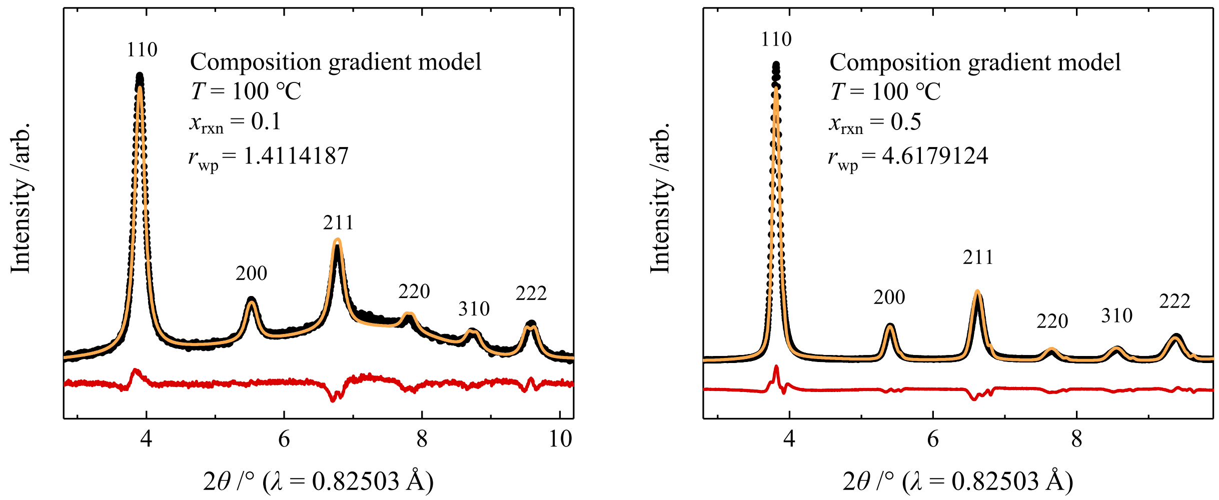

Figure S9: Composition gradient fits to high-resolution XRD data for samples synthesised at $T = 100\,°\mathrm{C}$; $x_{\mathrm{rxn}} = 0.1$ and 0.5. Observed, calculated and difference data are shown as black dots, and orange and red lines, respectively.

S3 Solid state nuclear magnetic resonance

Solid state MAS NMR spectra were obtained for $Zn_{0.5}Cd_{0.5}(mIm)_2$ samples synthesised at 40 °C, 60 °C, 80 °C, and 100 °C (Figure S10). The aromatic ^{13}C signals, while sensitive to the metals present, do not provide any information on ordering, with three very closely-spaced resonances for the CH species at 124.9, 124.6 and 124.2 ppm and a single quaternary resonance at 151.4 ppm. The latter is mid-way between 151.1 ppm for the Zn end-member and 151.7 ppm for the Cd end-member. Unfortunately, the CH resonances are not sufficiently resolved to allow unambiguous decomposition and integration of the signal, especially since there is a poorly-defined broad resonance in the baseline of all four samples, centred at ~126 ppm, which further complicates the deconvolution. The CH_3 resonances (inset, Figure S10) are better resolved and, in all cases, have three components at 13.7, 14.0 and 14.4 ppm. However, interpretation of these signals is complicated by issues with decomposition (including a broader, low-intensity resonance at ~14.6 ppm) and the fact that the chemical shifts of the CH_3 groups in these ZIFs are more sensitive to disorder and magnetic (*i.e.*, crystallographic) inequivalence than to the nature of the metal atoms three bonds away. Therefore, in this case, it is impossible to assign these resonances directly to differences in chemistry or structure.

The ^{15}N NMR spectra (Figure S10, right), are far more sensitive to the nature of the N-bound metal. In this case, broadened ^{15}N resonances are observed at –173.5(2) ppm (corresponding to N-Cd) and –176.3(2) ppm (N-Zn) for the mixed-metal ZIFs. In addition, a minor resonance at –171 ppm can be attributed to one half of the $^{111/113}Cd$-^{15}N J-coupled doublet ($J \simeq 200$ Hz), with the other half overlapping with the N-Zn signal. The spectra are sufficiently resolved that they can be integrated to obtain approximate Cd/Zn ratios. These ratios are plotted in Figure S11 and it can be seen that the Cd content of the product decreases significantly with increasing synthesis temperature, as found by XRD and STEM–EDS. We note that, in this case, it is not possible to use ^{15}N NMR spectroscopy to investigate the ordering of the metal cations.

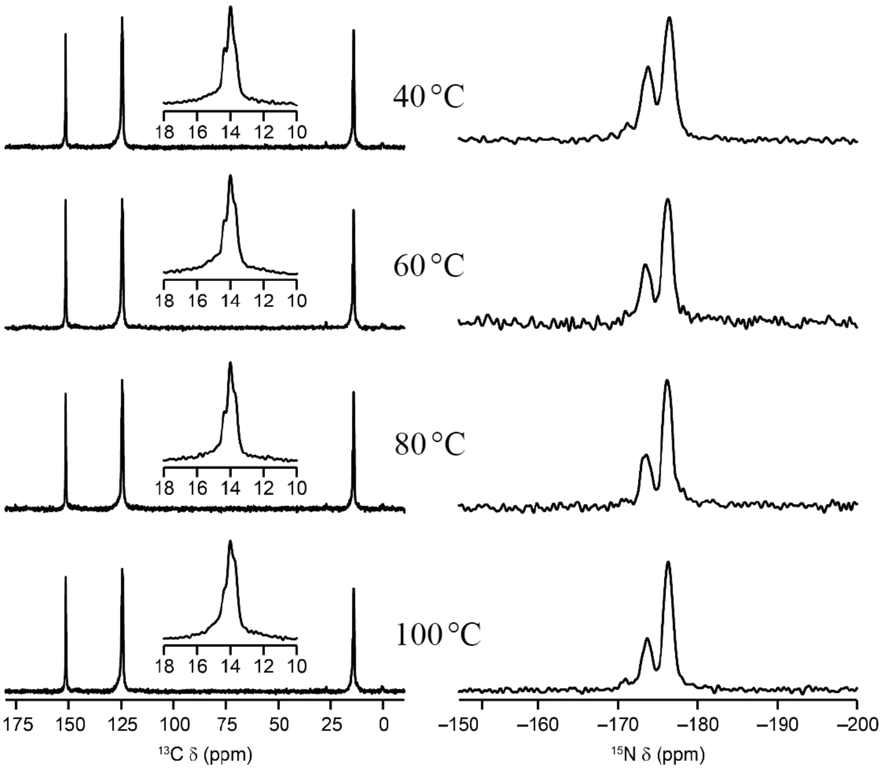

Figure S10: Solid state MAS NMR spectra (^{13}C, left; ^{15}N, right) of Zn/Cd ZIF-8 samples synthesised at 40 °C, 60 °C, 80 °C, and 100 °C with reaction Cd mole fraction $x_{\mathrm{rxn}} = 0.5$.

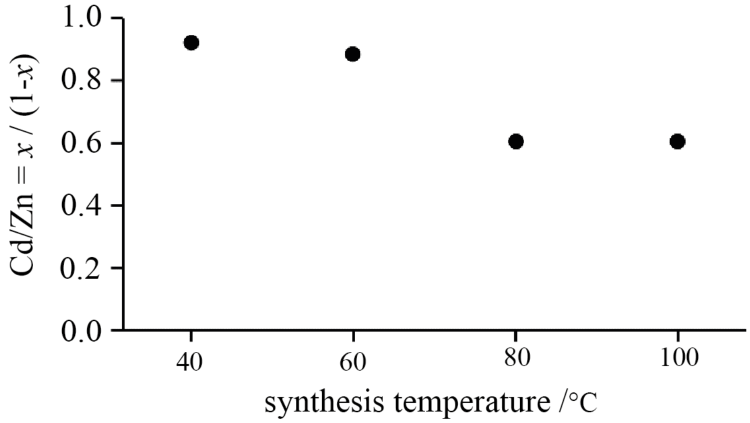

Figure S11: Cd/Zn ratios derived from integrated intensities of the ^{15}N NMR spectra shown in Figure S10.

S4 Additional STEM–EDS images

S4.1 STEM–EDS images and line profiles

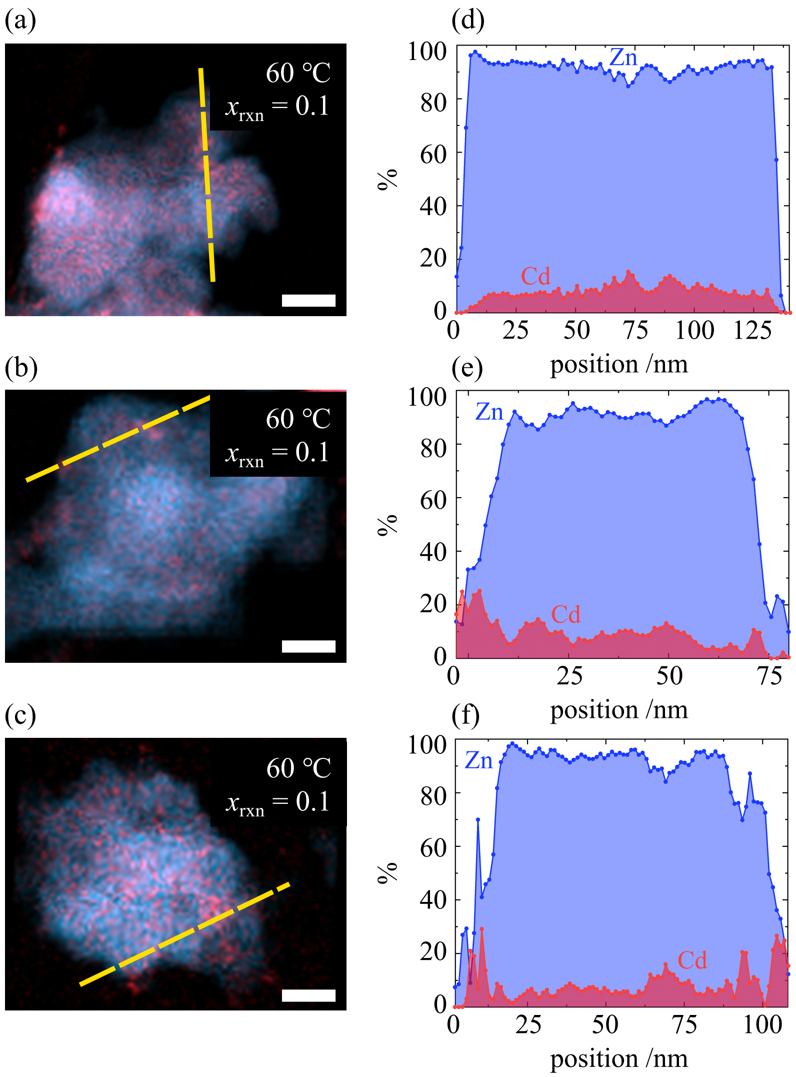

Figure S12: STEM–EDS images of Zn/Cd ZIF-8 nanoparticles synthesised at 60 °C (a–c), with reaction Cd mole fraction $x_{rxn} = 0.1$. Line profiles (d–f), corresponding to dashed lines in (a–c), respectively, show the percentage of Zn and Cd at each pixel. Scale bars = 30 nm.

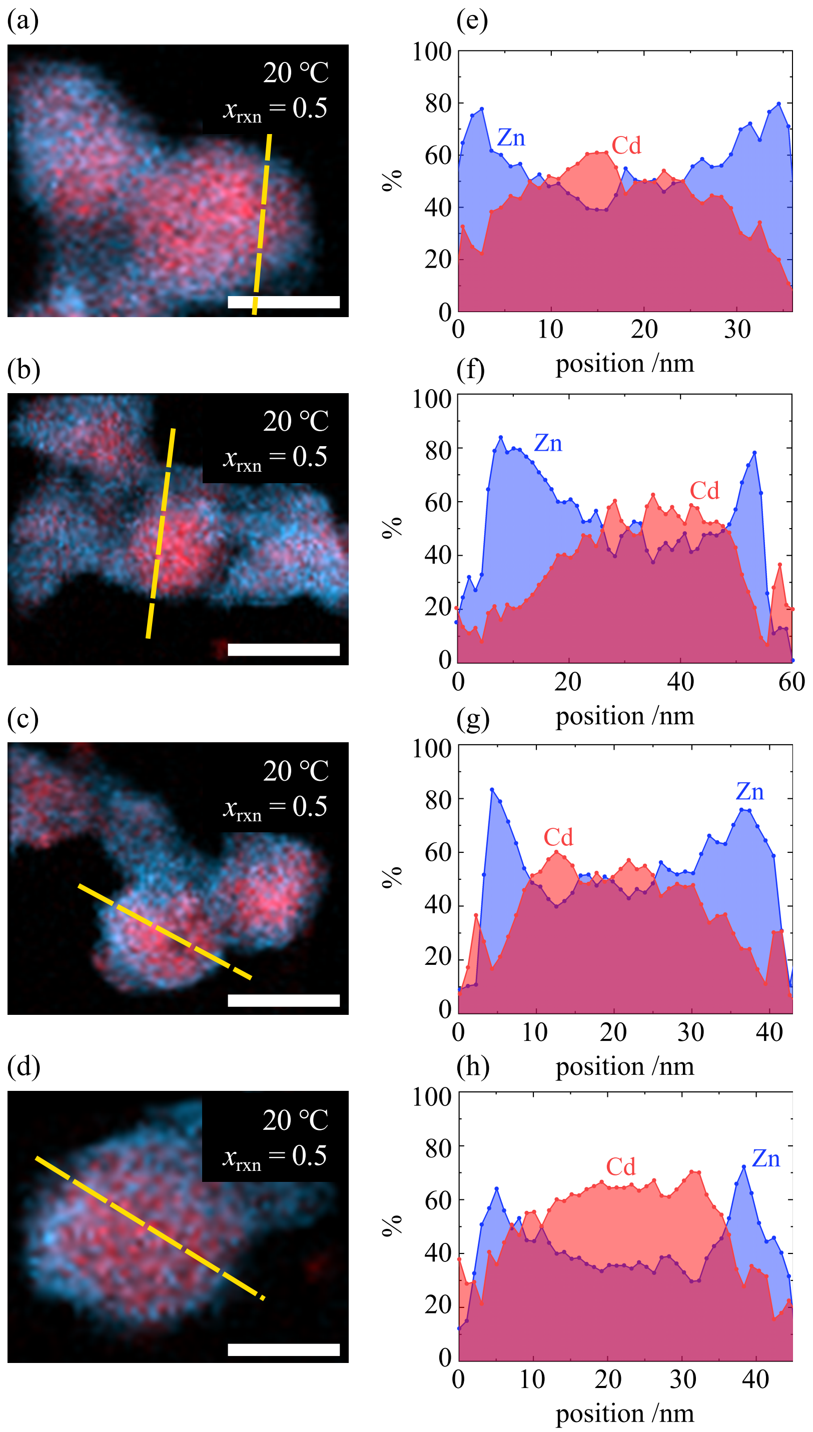

Figure S13: STEM–EDS images of Zn/Cd ZIF-8 nanoparticles synthesised at 20 °C (a–d), with reaction Cd mole fraction $x_{\mathrm{rxn}} = 0.5$. Line profiles (e–h), corresponding to dashed lines in (a–d), respectively, show the percentage of Zn and Cd at each pixel. Scale bars = 30 nm.

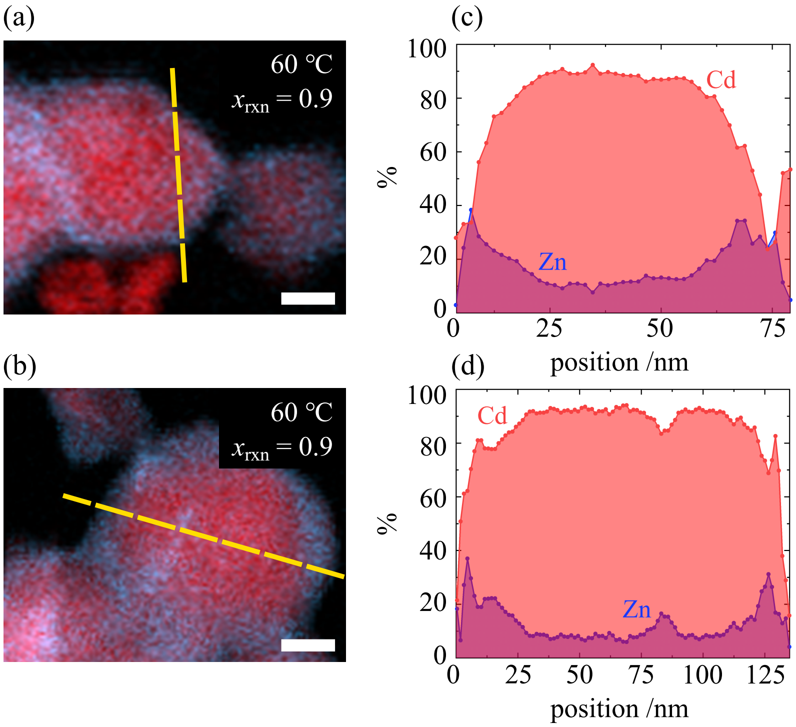

Figure S14: STEM–EDS images of Zn/Cd ZIF-8 nanoparticles synthesised at 60 °C (a–b), with reaction Cd mole fraction $x_{\mathrm{rxn}} = 0.9$. Line profiles (c–d), corresponding to dashed lines in (a–b), respectively, show the percentage of Zn and Cd at each pixel. Scale bars = 30 nm.

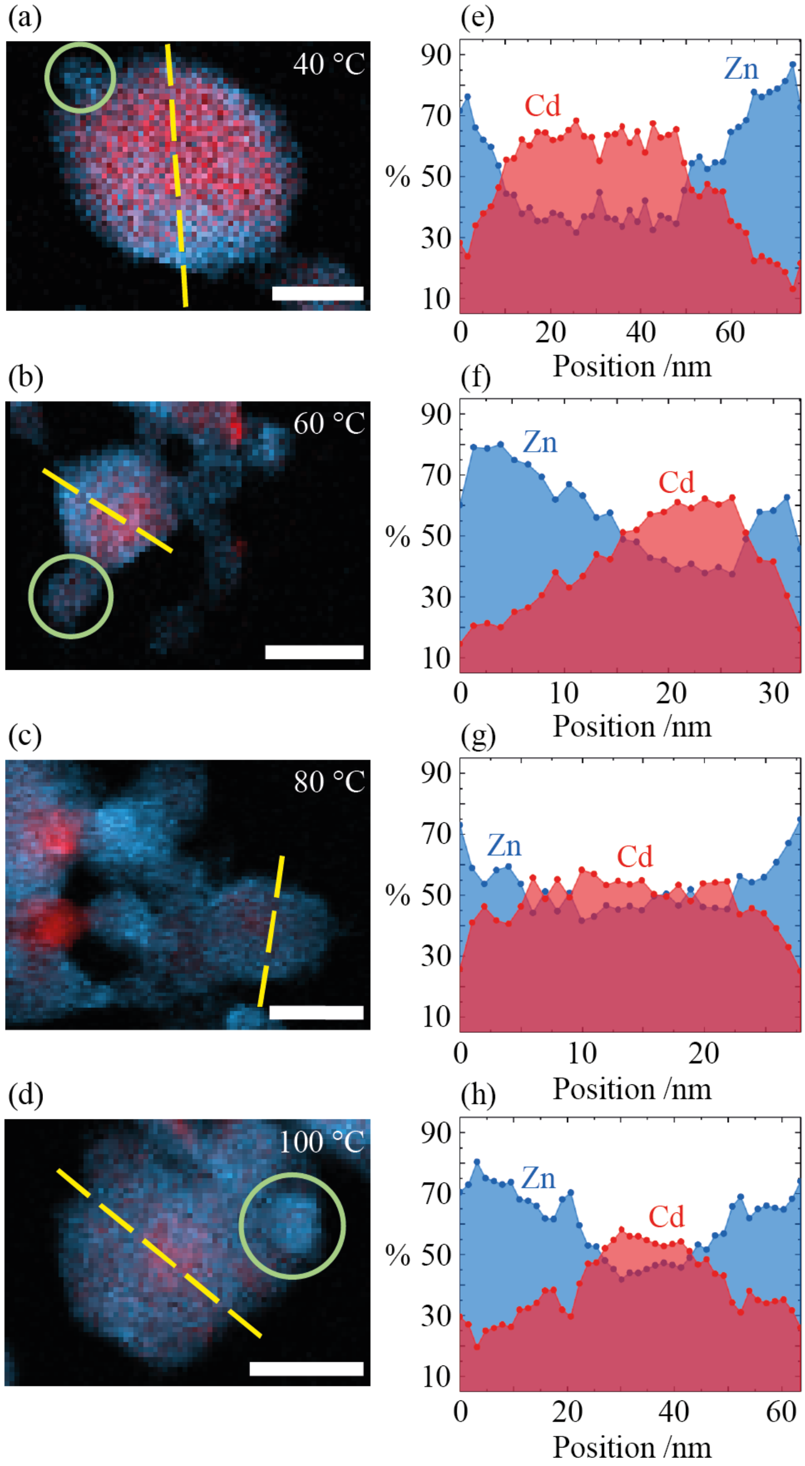

Figure S15: STEM–EDS images of Zn/Cd ZIF-8 nanoparticles synthesised at 40 °C, 60 °C, 80 °C, and 100 °C (a–d), respectively, with reaction Cd mole fraction $x_{\mathrm{rxn}} = 0.5$. Line profiles (e–h), corresponding to dashed lines in (a–d), respectively, show the percentage of Zn and Cd at each pixel. Scale bars = 30 nm. The green circles in (a), (b) and (d) indicate Zn-rich nanoparticles possibly formed by secondary nucleation.

S4.2 Individual ADF images, EDS maps and composite images

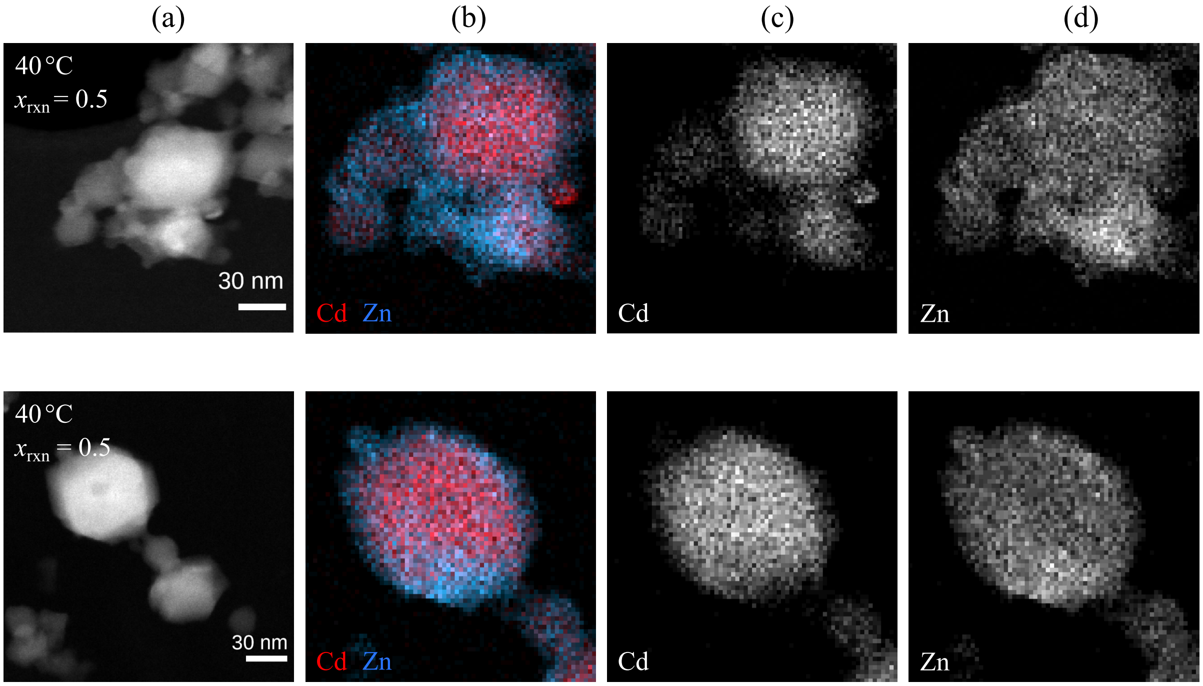

Figure S16: STEM images of Zn/Cd ZIF-8 nanoparticles synthesised at 40 °C with reaction Cd mole fraction $x_{\text{rxn}} = 0.5$, showing (a) annular dark field images, (b) combined Zn-Cd EDS maps, (c) individual Cd EDS maps and (d) individual Zn EDS maps. Scale bars = 30 nm.

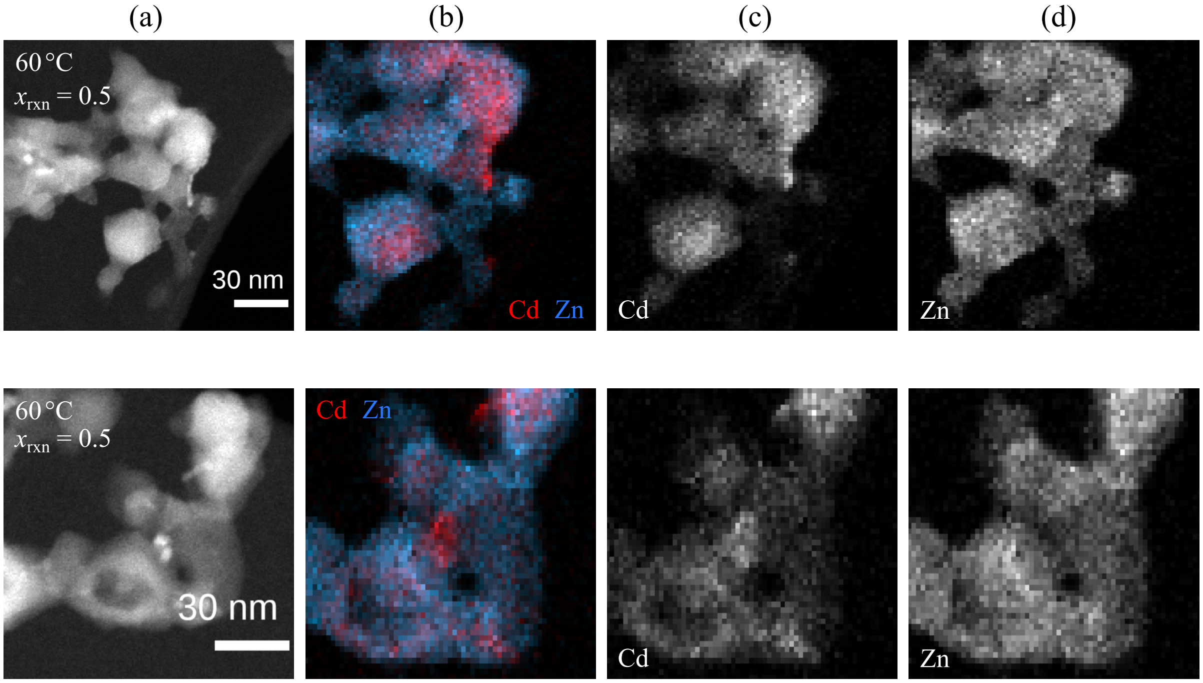

Figure S17: STEM images of Zn/Cd ZIF-8 nanoparticles synthesised at 60 °C with reaction Cd mole fraction $x_{\text{rxn}} = 0.5$, showing (a) annular dark field images, (b) combined Zn-Cd EDS maps, (c) individual Cd EDS maps and (d) individual Zn EDS maps. Scale bars = 30 nm.

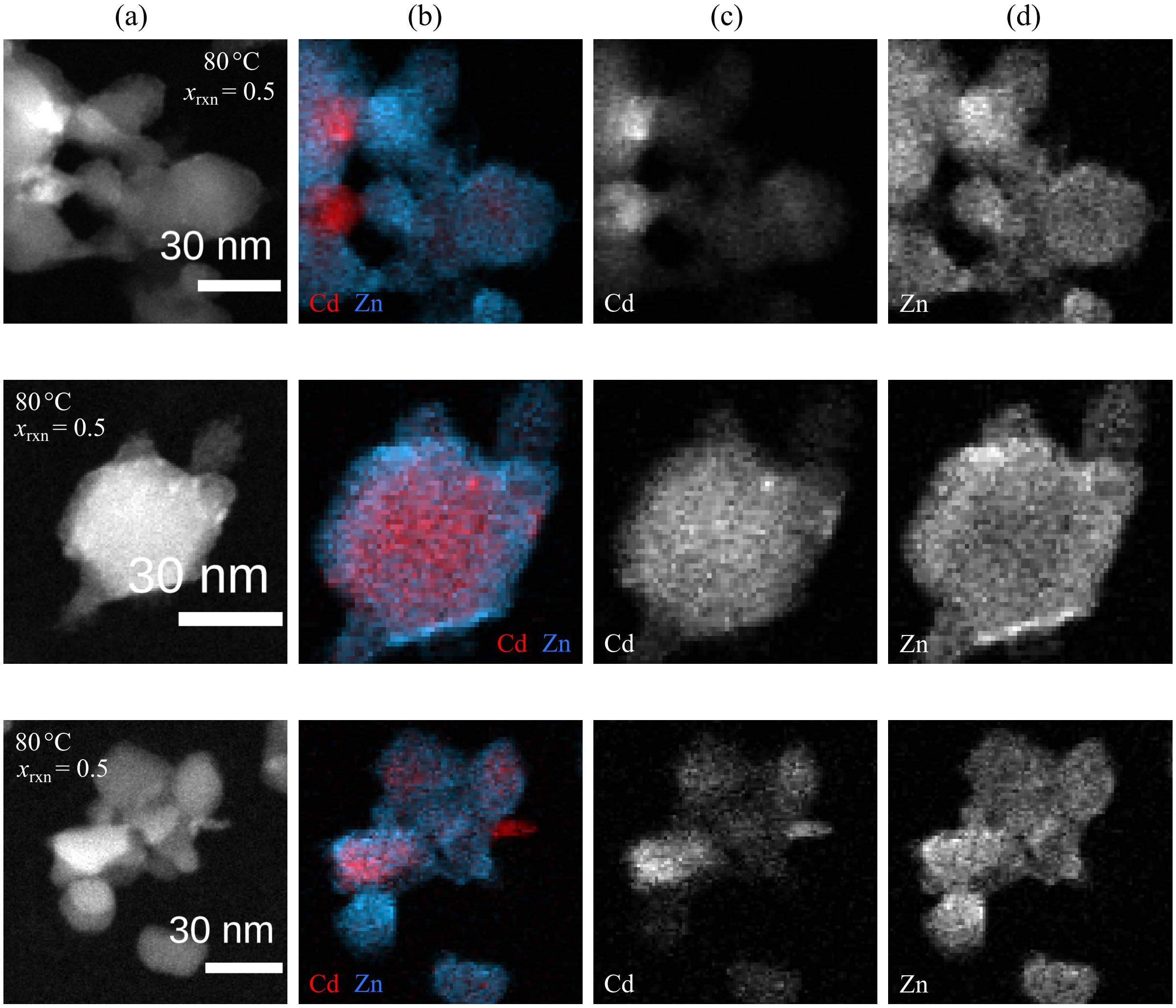

Figure S18: STEM images of Zn/Cd ZIF-8 nanoparticles synthesised at 80 °C with reaction Cd mole fraction $x_{\text{rxn}} = 0.5$, showing (a) annular dark field images, (b) combined Zn-Cd EDS maps, (c) individual Cd EDS maps and (d) individual Zn EDS maps. Scale bars = 30 nm.

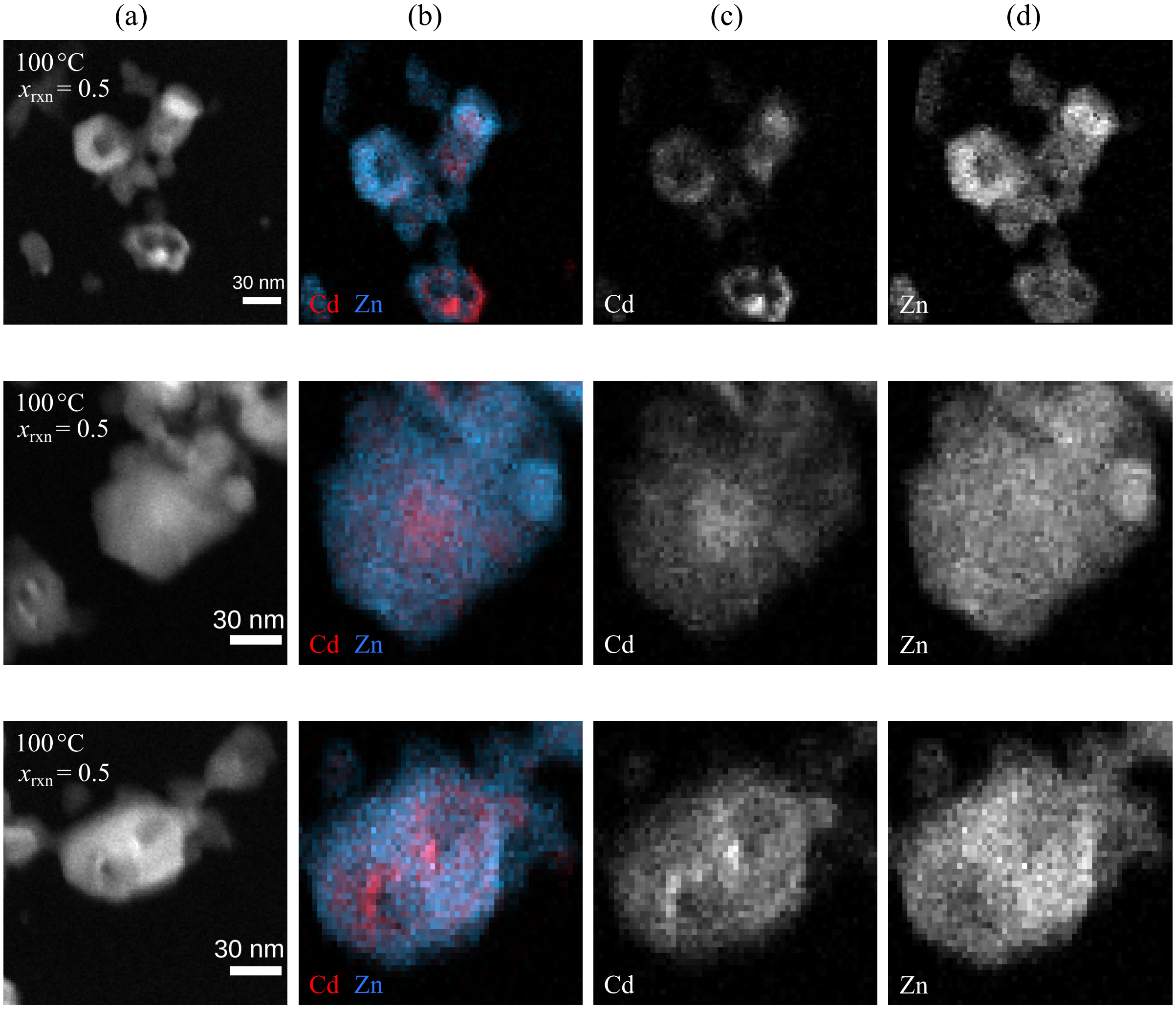

Figure S19: STEM images of Zn/Cd ZIF-8 nanoparticles synthesised at 100 °C with reaction Cd mole fraction $x_{\mathrm{rxn}} = 0.5$, showing (a) annular dark field images, (b) combined Zn-Cd EDS maps, (c) individual Cd EDS maps and (d) individual Zn EDS maps. Scale bars = 30 nm.

S4.3 Particle size distributions

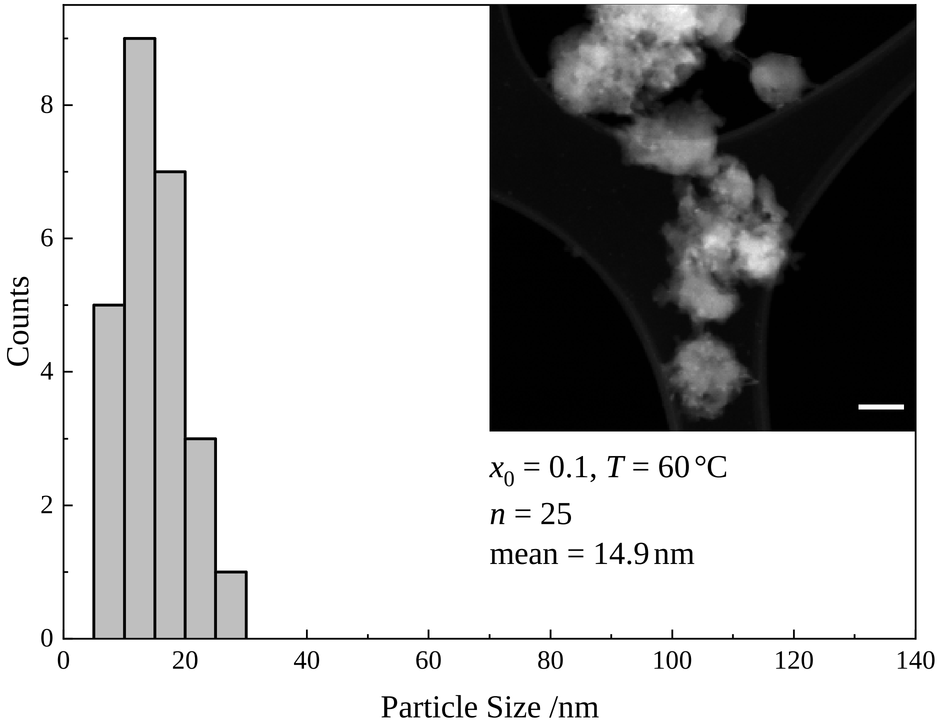

Figure S20: Particle size distribution of Zn/Cd ZIF-8 nanoparticles synthesised at 60 °C with reaction Cd mole fraction $x_{\mathrm{rxn}} = 0.1$, calculated from a total of 25 measurements. Inset shows the corresponding wide field of view annular dark field image, in which aggregation of relative fine primary grains, as observed in the EDS, can clearly be seen. Scale bar = 75 nm.

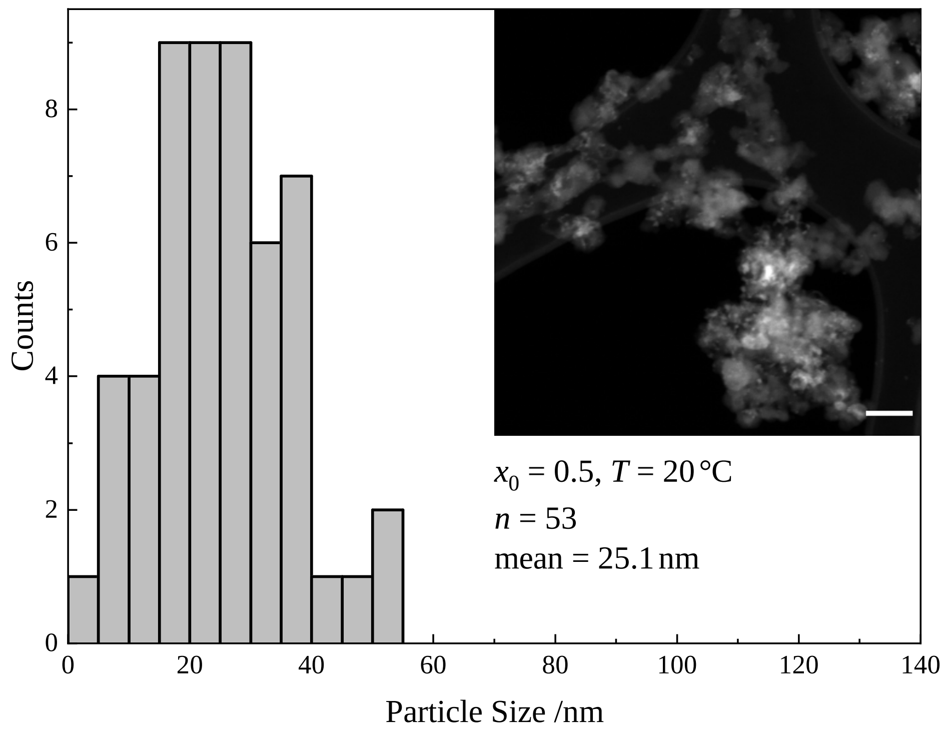

Figure S21: Particle size distribution of Zn/Cd ZIF-8 nanoparticles synthesised at 20 °C with reaction Cd mole fraction $x_{\mathrm{rxn}} = 0.5$, calculated from a total of 53 measurements. Inset shows the corresponding wide field of view annular dark field image. Scale bar = 75 nm.

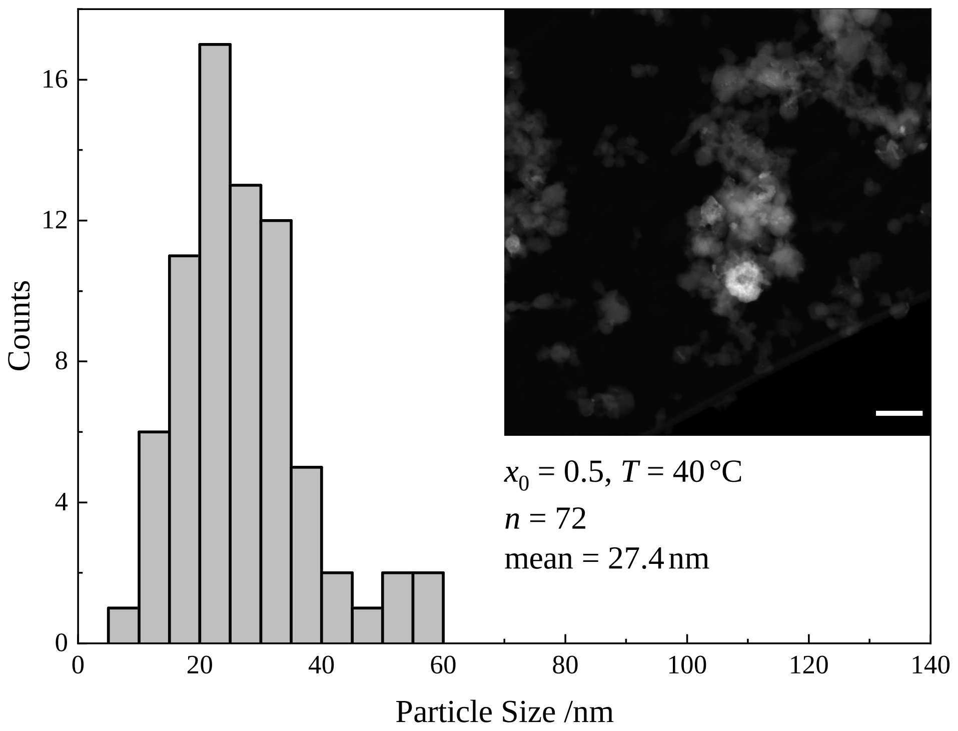

Figure S22: Particle size distribution of Zn/Cd ZIF-8 nanoparticles synthesised at 40 °C with reaction Cd mole fraction $x_{\mathrm{rxn}} = 0.5$, calculated from a total of 72 measurements. Inset shows the corresponding wide field of view annular dark field image. Scale bar = 75 nm.

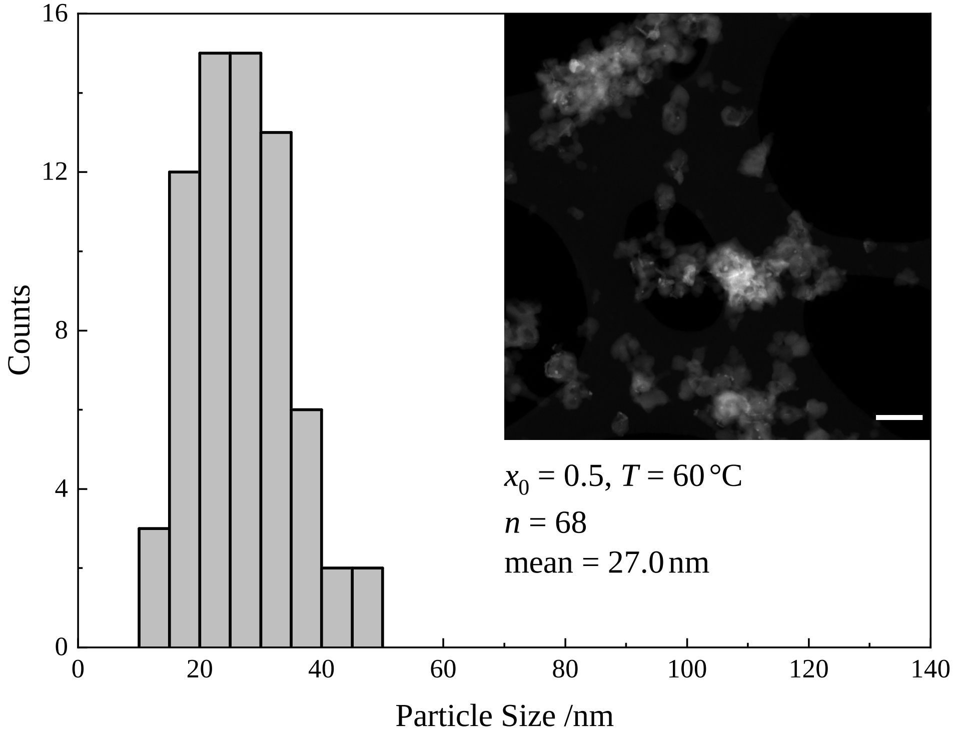

Figure S23: Particle size distribution of Zn/Cd ZIF-8 nanoparticles synthesised at 60 °C with reaction Cd mole fraction $x_{\mathrm{rxn}} = 0.5$, calculated from a total of 68 measurements. Inset shows the corresponding wide field of view annular dark field image. Scale bar = 75 nm.

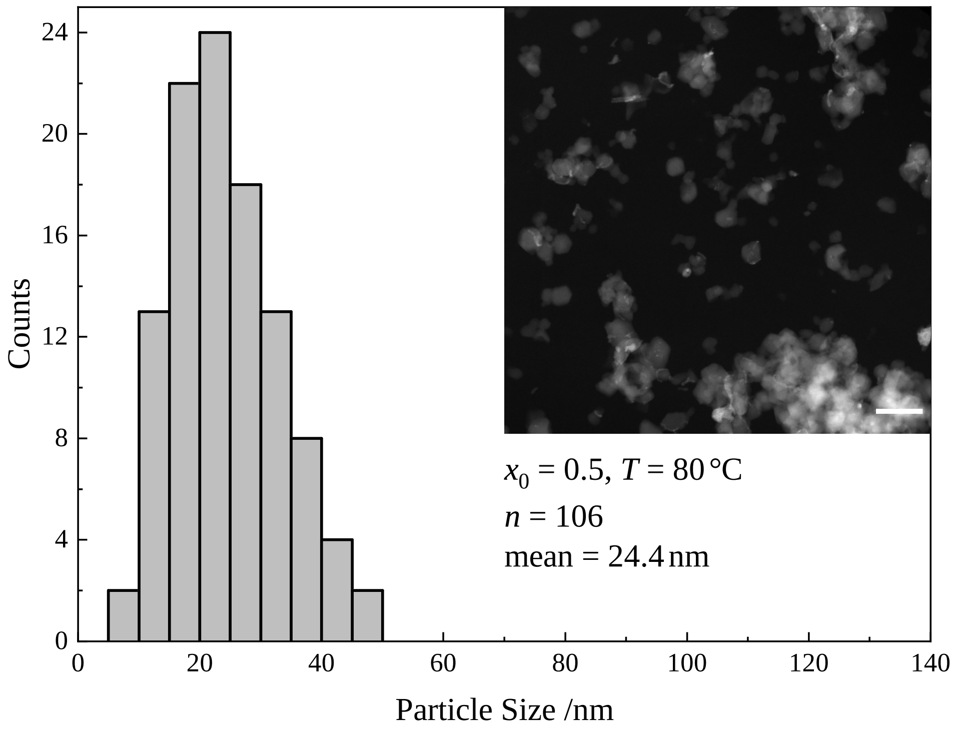

Figure S24: Particle size distribution of Zn/Cd ZIF-8 nanoparticles synthesised at 80 °C with reaction Cd mole fraction $x_{\mathrm{rxn}} = 0.5$, calculated from a total of 106 measurements. Inset shows the corresponding wide field of view annular dark field image. Scale bar = 75 nm.

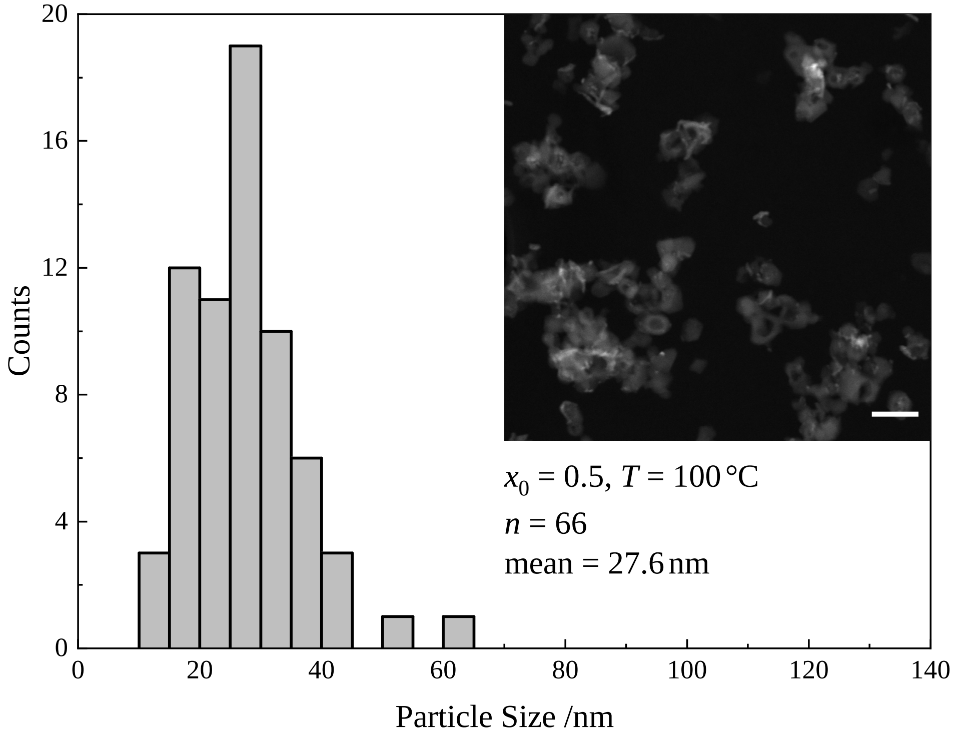

Figure S25: Particle size distribution of Zn/Cd ZIF-8 nanoparticles synthesised at 100 °C with reaction Cd mole fraction $x_{\mathrm{rxn}} = 0.5$, calculated from a total of 66 measurements. Inset shows the corresponding wide field of view annular dark field image. Scale bar = 75 nm.

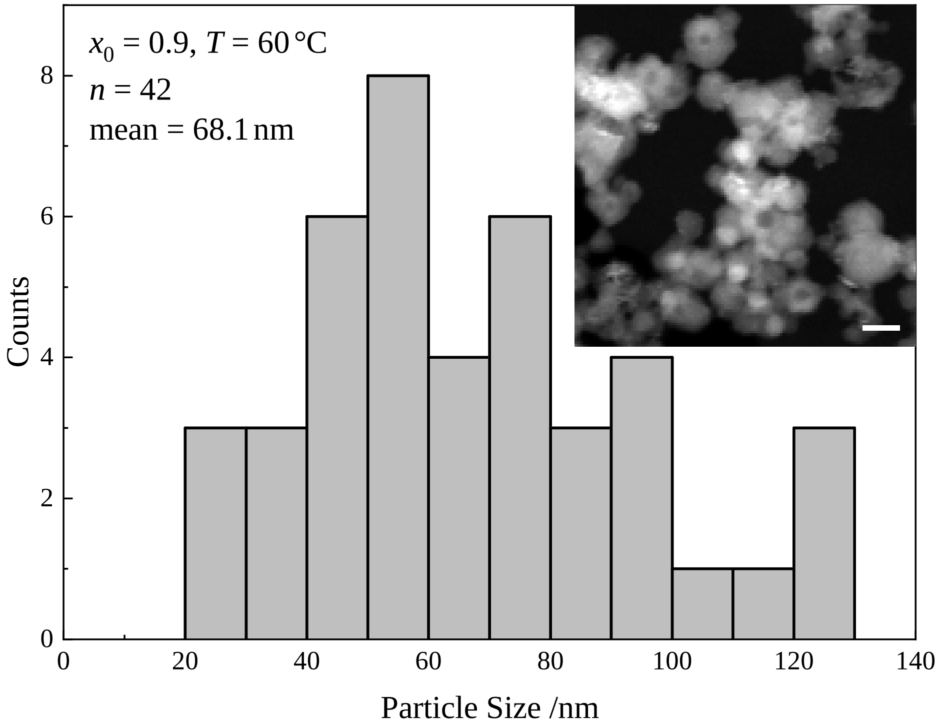

Figure S26: Particle size distribution of Zn/Cd ZIF-8 nanoparticles synthesised at 60 °C with reaction Cd mole fraction $x_{\mathrm{rxn}} = 0.9$, calculated from a total of 42 measurements. Inset shows the corresponding wide field of view annular dark field image. Scale bar = 75 nm.

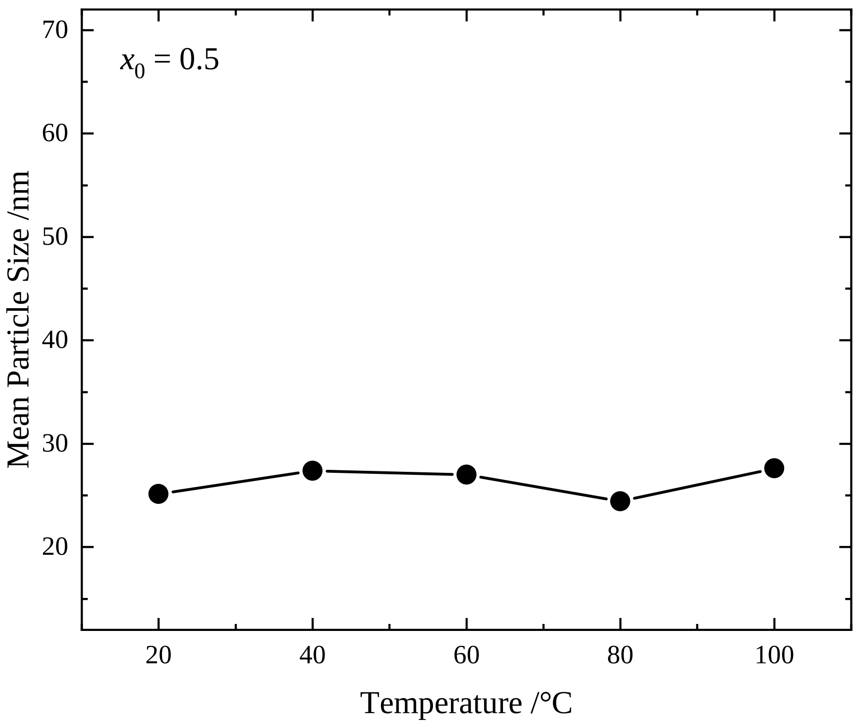

Figure S27: Variation in mean particle size as a function of synthesis temperature, T, for Zn/Cd ZIF-8 nanoparticles with reaction Cd mole fraction $x_{\rm rxn} = 0.5$.

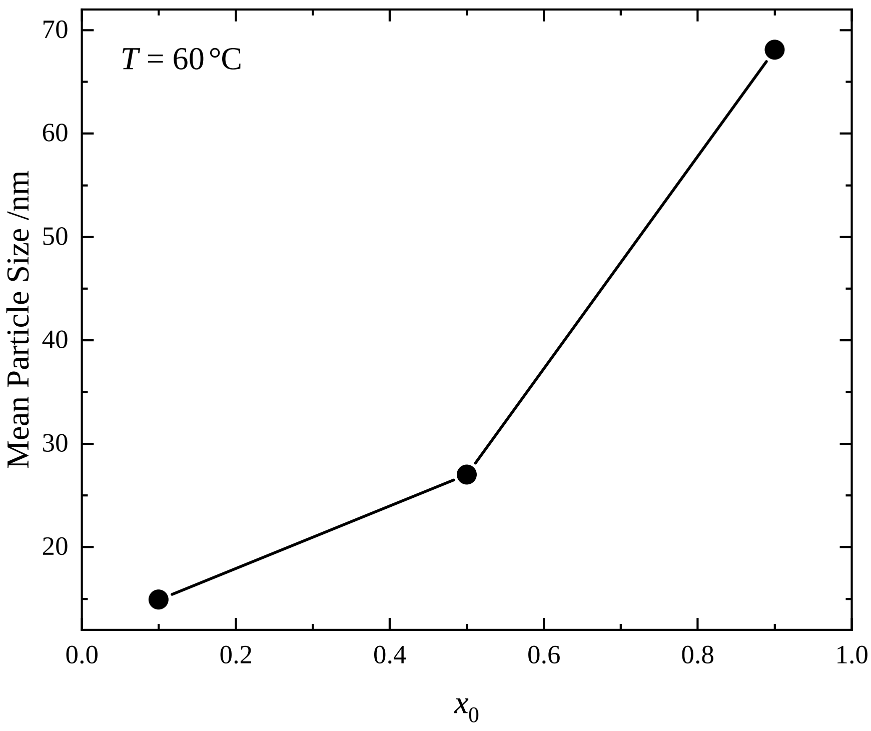

Figure S28: Variation in mean particle size as a function of reaction Cd mole fraction, x_{rxn}, for Zn/Cd ZIF-8 nanoparticles synthesised at 60 °C.

S5 Radial composition profiles for all samples

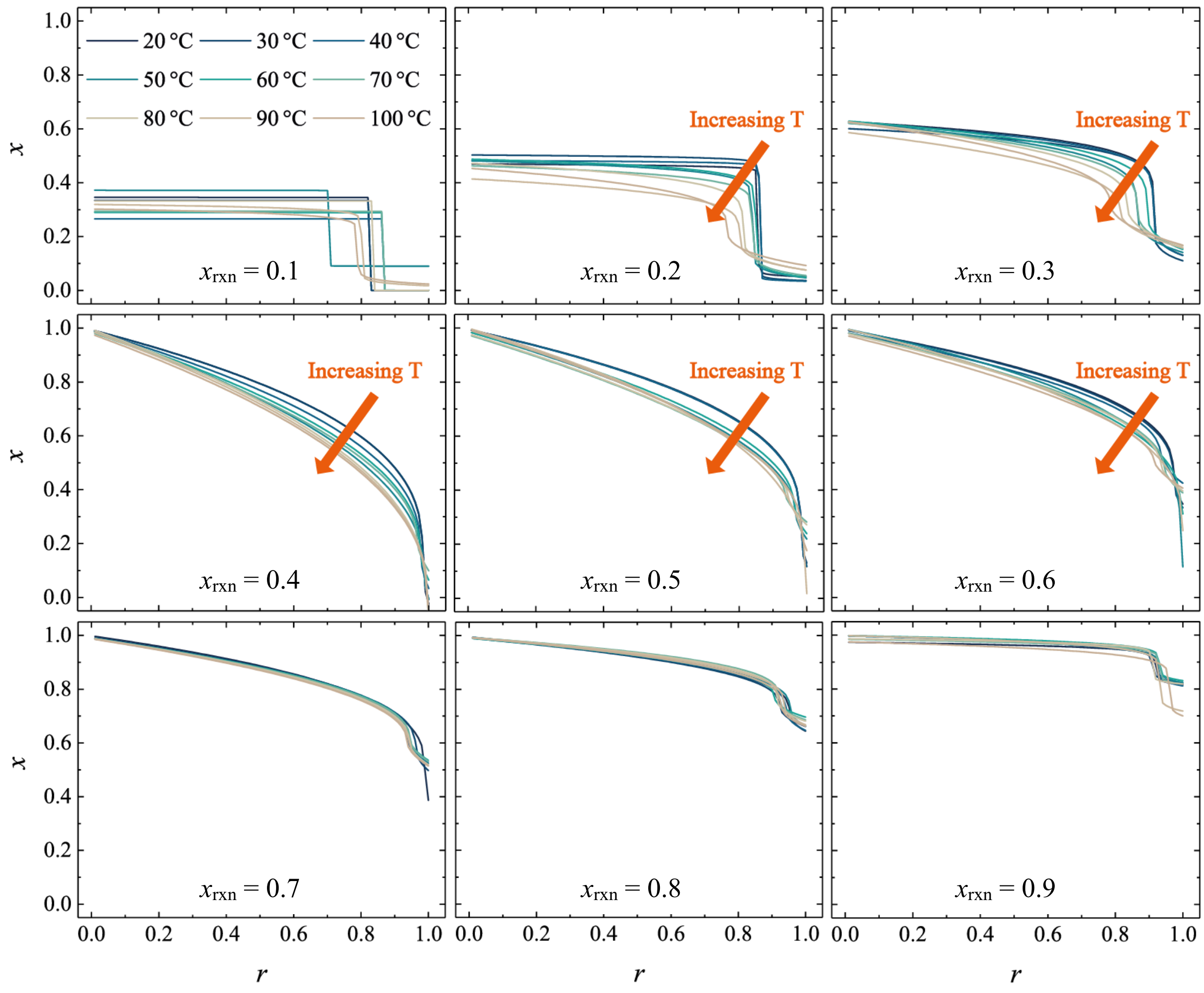

Figure S29: Radial composition profiles for Zn/Cd ZIF-8 synthesised at different temperatures and with different initial mole fractions of Cd, x_{rxn}. The orange arrows indicate how these functions change with increasing synthesis temperature for five of the series where the trend is most apparent. $r = 0$ is the centre of the nanoparticle and $r = 1$ is the edge of the nanoparticle.

S6 Synthesis–structure prediction maps relating two-phase model refinements

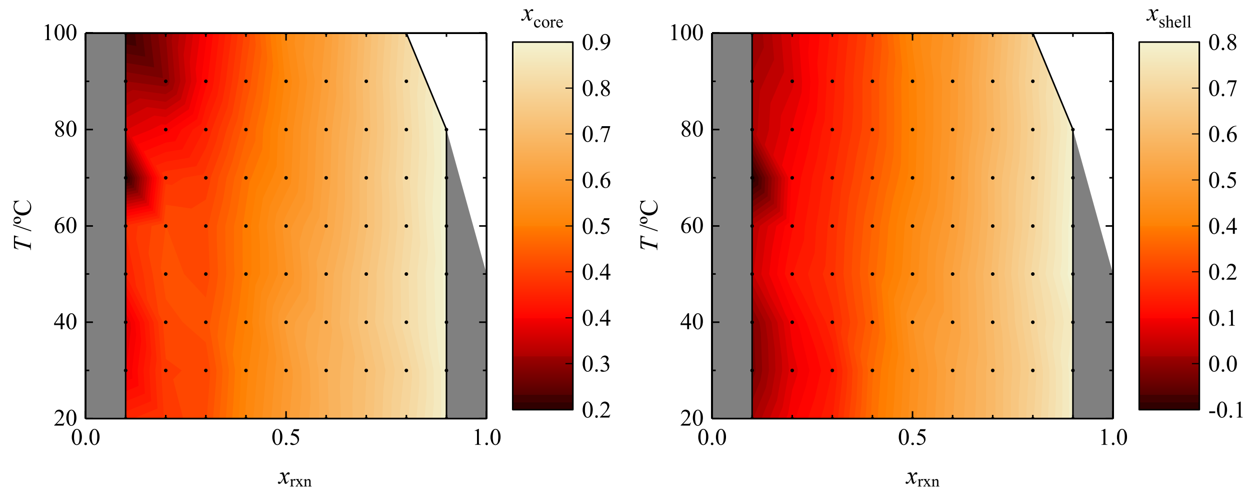

Figure S30: Variation of Cd mole fractions of the two phases, denoted x_{core} and x_{shell}, with synthesis conditions determined using two-phase refinements of high-resolution synchrotron XRD data. Note the different scales. Grey regions correspond to the pure end-members for which a two-phase refinement is not physically meaningful. The white region indicates conditions outside the synthesis window for ZIF-8; black dots indicate the data points.

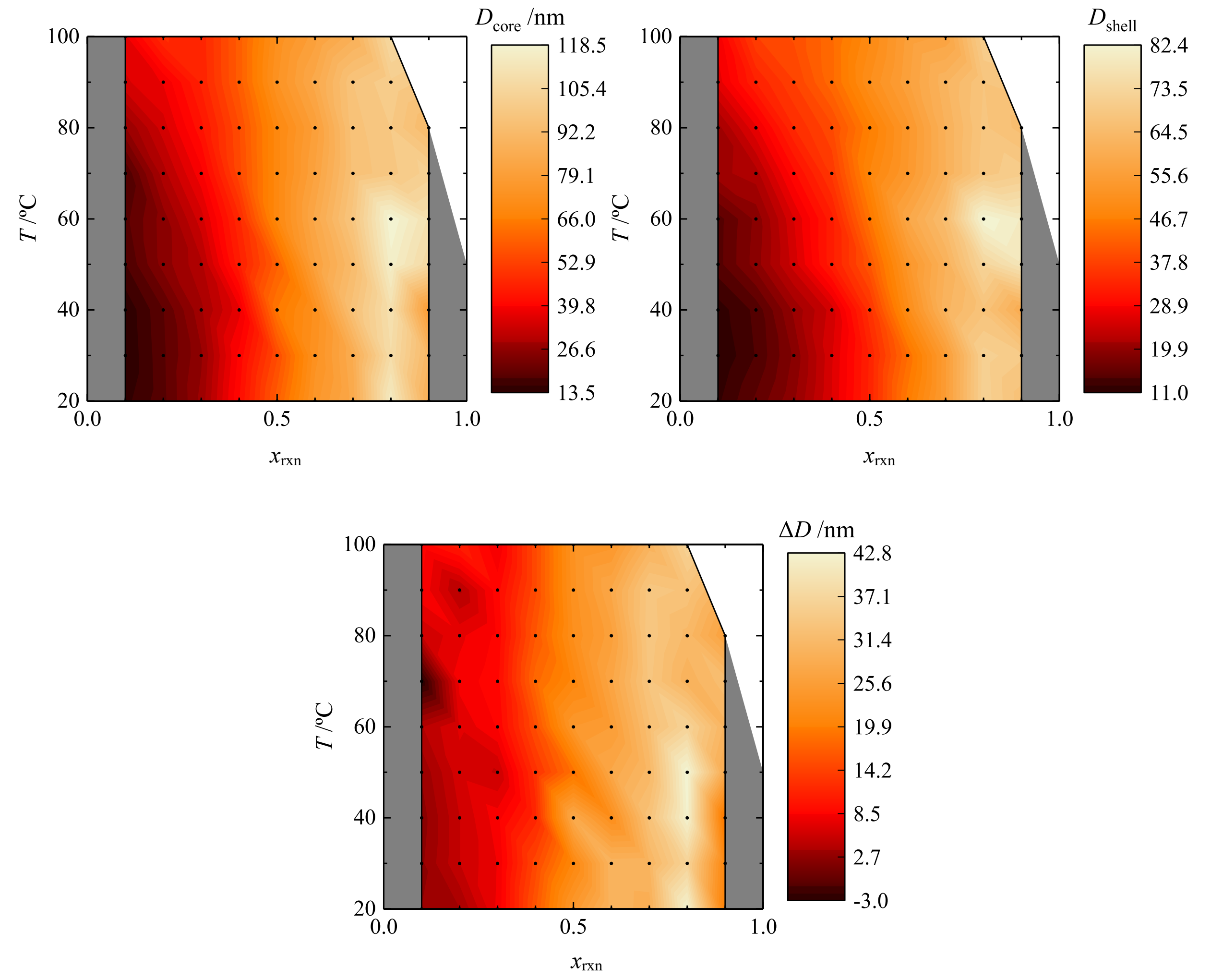

Figure S31: Variation of coherent scattering domain size of the two phases, denoted D_{core} and D_{shell}, and the difference between them, ΔD, with synthesis conditions determined using two-phase refinements of high-resolution synchrotron XRD data. Note the different scales. Grey regions correspond to the pure end-members for which a two-phase refinement is not physically meaningful. The white region indicates conditions outside the synthesis window for ZIF-8; black dots indicate the data points.

S7 Composition gradient refinement fits to *in situ* XRD data

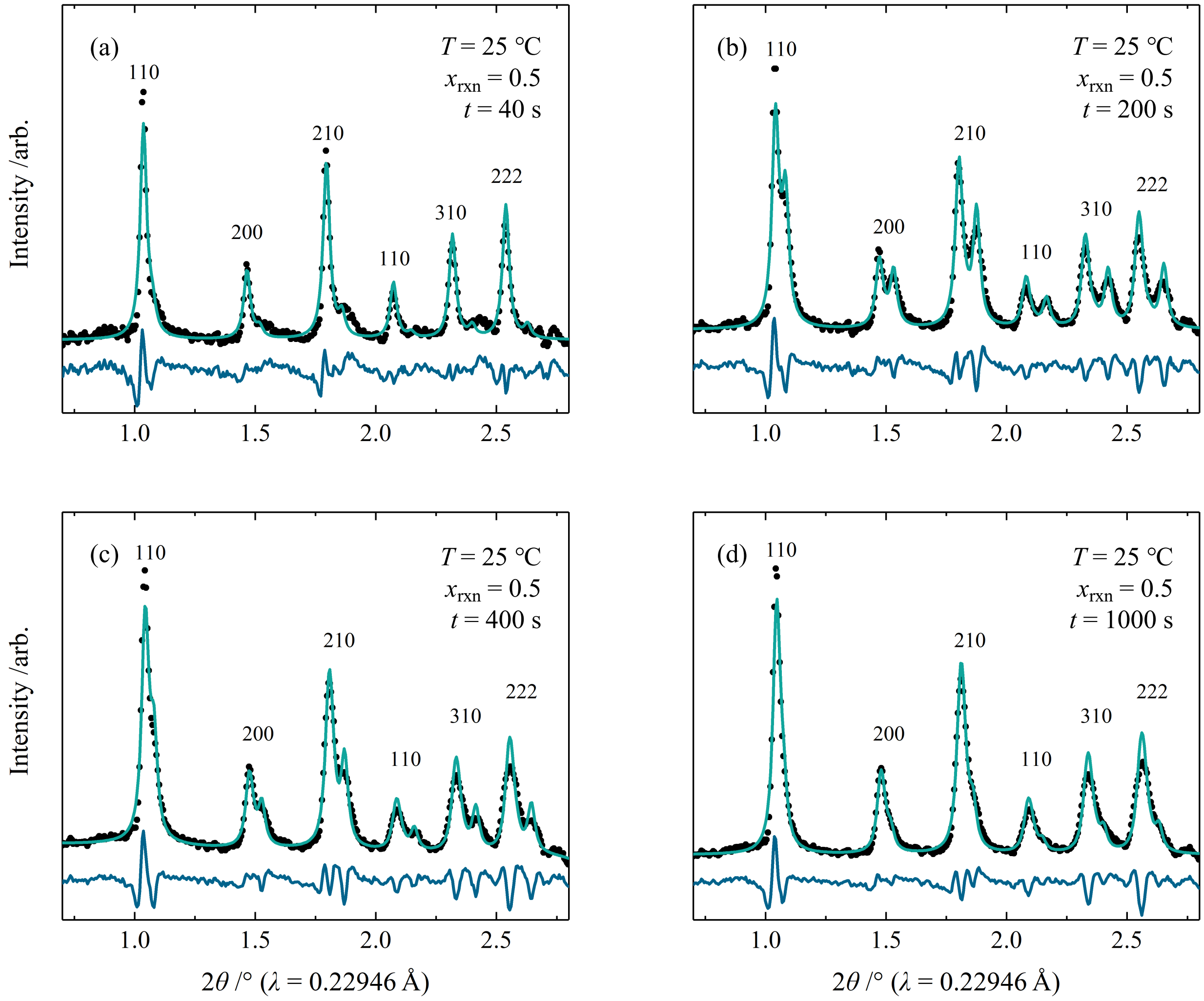

Figure S32: Composition gradient refinement fit to *in situ* XRD data of mixed Zn/Cd ZIF-8 crystallisation at 25 °C for (x_{rxn} = 0.5) at times t = 40 s, 200 s, 400 s and 1000 s ((a–d), respectively).

S8 *In situ* XRD data for Zn/Cd ZIF-8 crystallisation above 25 °C

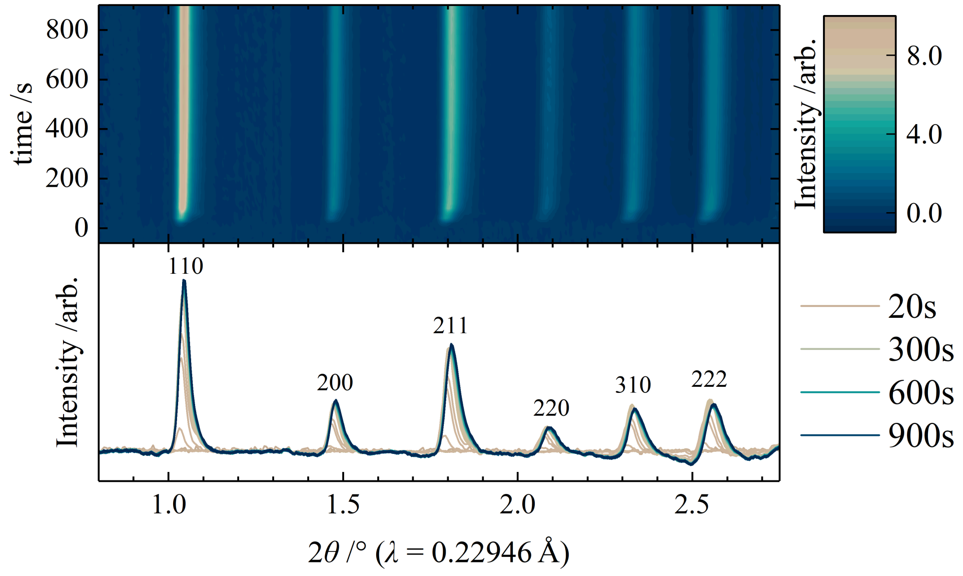

Figure S33: *In situ* synchrotron XRD data for mixed Zn/Cd ZIF-8 crystallisation at 35 °C, $x_{\mathrm{rxn}} = 0.5$. A rolling ball background subtraction has been performed for clarity.

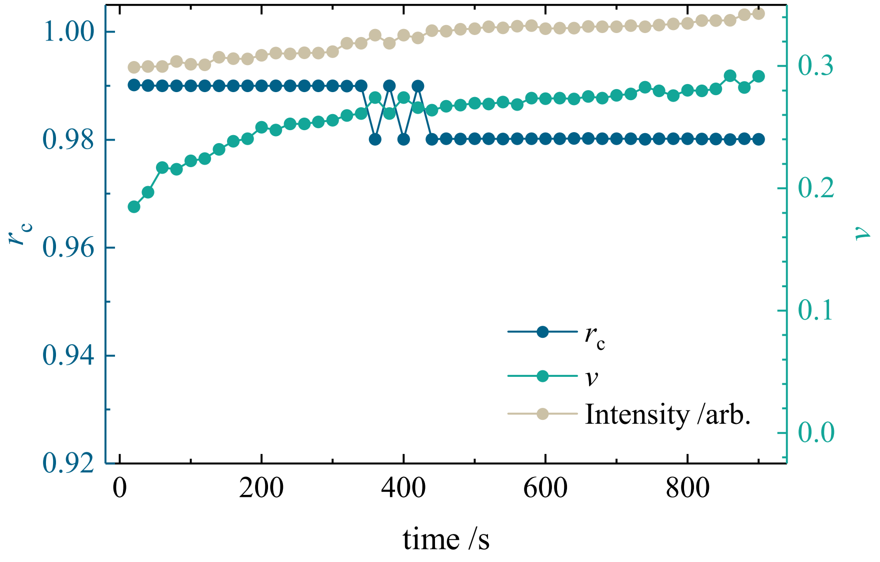

Figure S34: Temporal evolution of total Bragg peak intensity, I (beige), interface diffuseness, ν (turquoise), and nominal interface radius, r_c (blue) for mixed Zn/Cd ZIF-8 crystallisation at 35 °C, $x_{\mathrm{rxn}} = 0.5$, determined using composition gradient model fits to *in situ* XRD data.

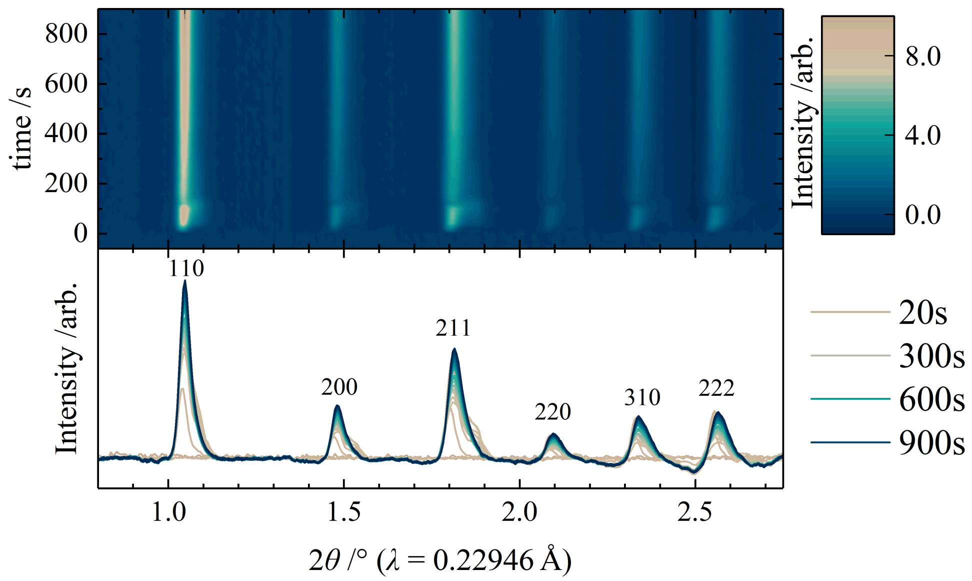

Figure S35: *In situ* synchrotron XRD data for mixed Zn/Cd ZIF-8 crystallisation at 45 °C, $x_{rxn} = 0.5$. A rolling ball background subtraction has been performed for clarity.

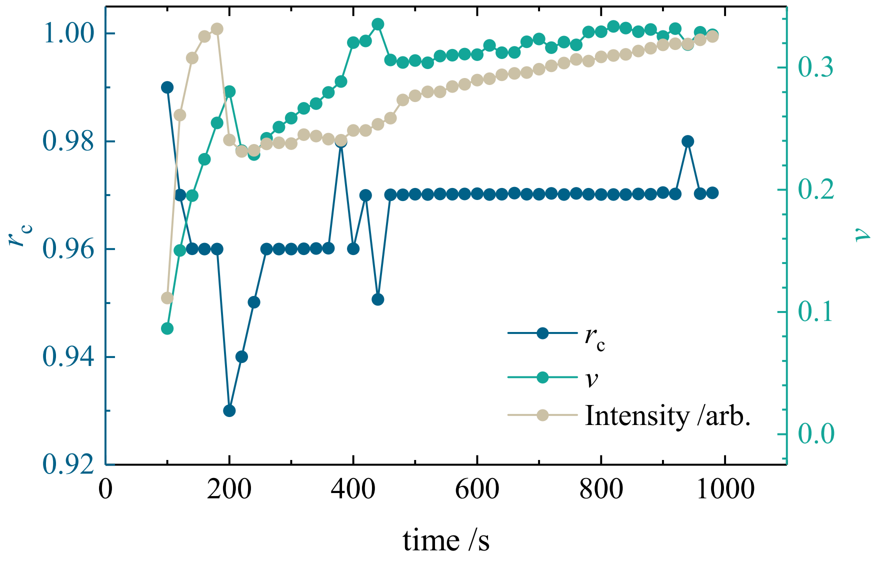

Figure S36: Temporal evolution of total Bragg peak intensity, I (beige), interface diffuseness, ν (turquoise), and nominal interface radius, r_c (blue) for mixed Zn/Cd ZIF-8 crystallisation at 45 °C, $x_{rxn} = 0.5$, determined using composition gradient model fits to *in situ* XRD data. The discontinuities around $t = 180$ s could not be accounted for in data processing and may be due to product falling out of the beam path.

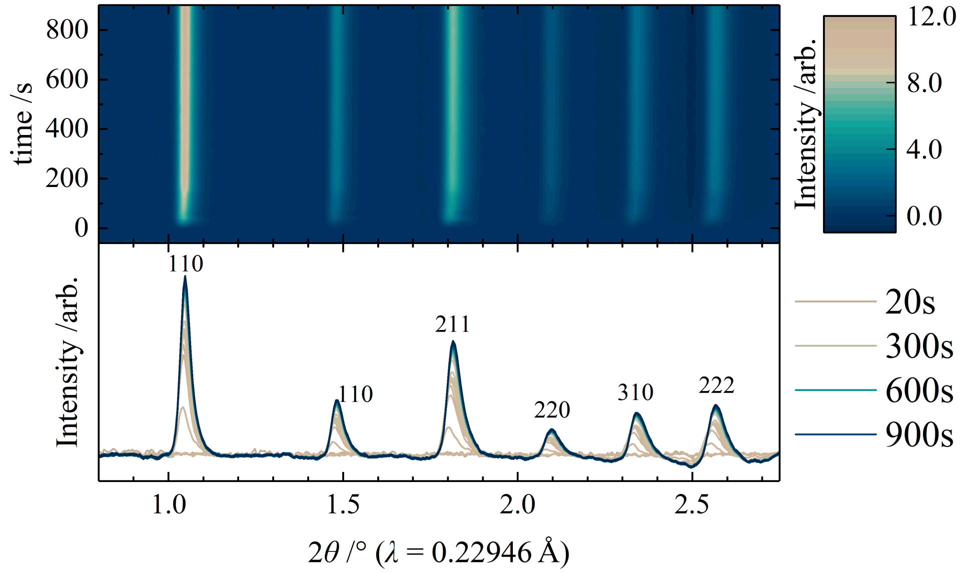

Figure S37: *In situ* synchrotron XRD data for mixed Zn/Cd ZIF-8 crystallisation at 55 °C, $x_{\mathrm{rxn}} = 0.5$. A rolling ball background subtraction has been performed for clarity.

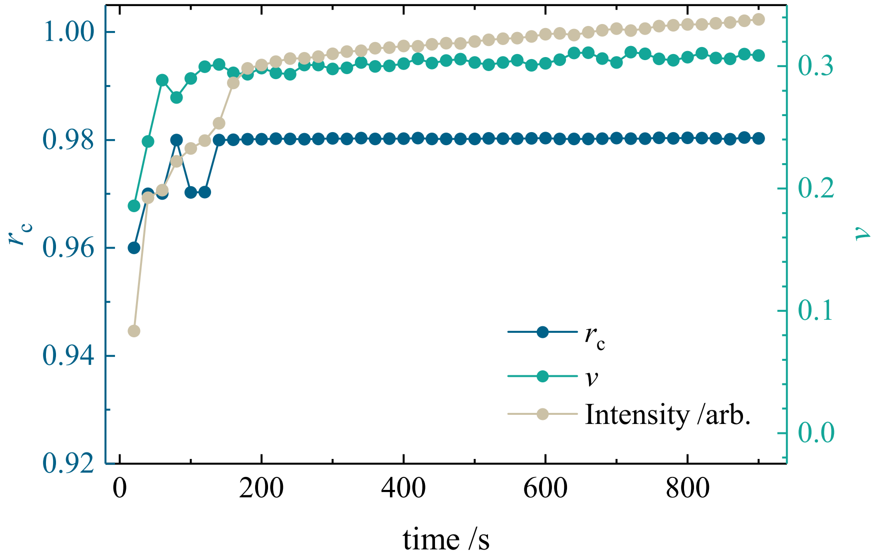

Figure S38: Temporal evolution of total Bragg peak intensity, I (beige), interface diffuseness, ν (turquoise), and nominal interface radius, r_c (blue) for mixed Zn/Cd ZIF-8 crystallisation at 55 °C, $x_{\mathrm{rxn}} = 0.5$, determined using composition gradient model fits to *in situ* XRD data.

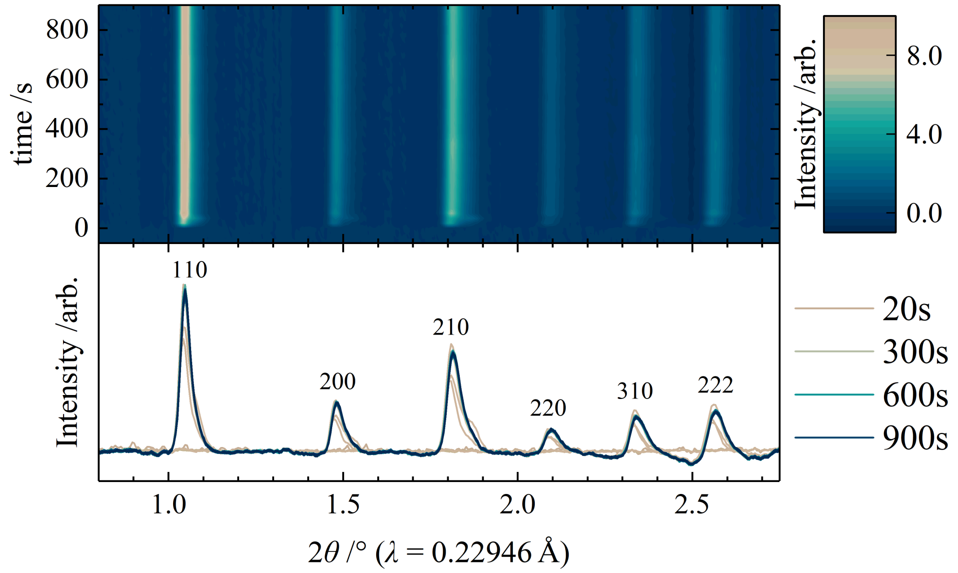

Figure S39: *In situ* synchrotron XRD data for mixed Zn/Cd ZIF-8 crystallisation at 65 °C, $x_{\mathrm{rxn}} = 0.5$. A rolling ball background subtraction has been performed for clarity.

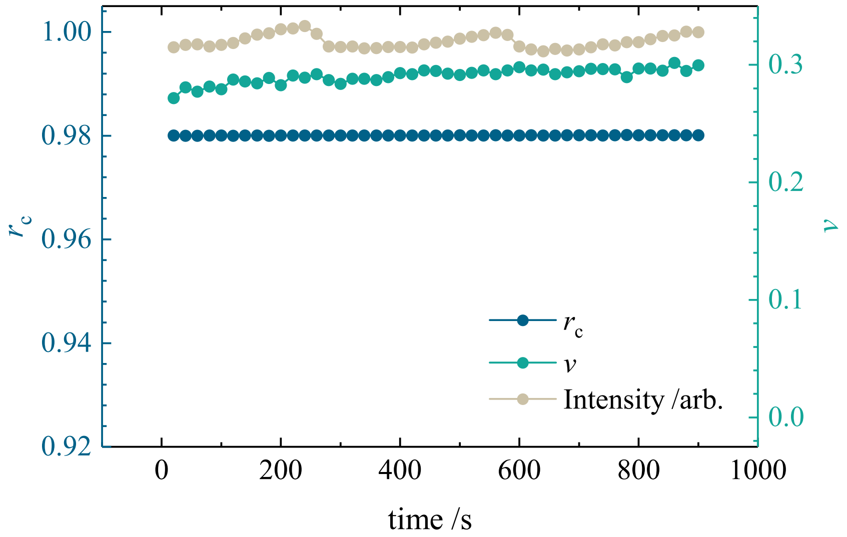

Figure S40: Temporal evolution of total Bragg peak intensity, I (beige), interface diffuseness, ν (turquoise), and nominal interface radius, r_{c} (blue) for mixed Zn/Cd ZIF-8 crystallisation at 65 °C, $x_{\mathrm{rxn}} = 0.5$, determined using composition gradient model fits to *in situ* XRD data. Note that the oscillations in Bragg peak intensity could not be accounted for in data processing and may be due to product falling in and out of the path of the beam.

S9 Kinetic analysis of parent Zn- and Cd-ZIF-8 crystallisation from *in situ* XRD data

Total integrated peak intensity data were extracted by Pawley fitting of *in situ* synchrotron XRD for the parent Zn- and Cd-ZIF-8 materials under identical conditions used for the mixed-component syntheses, at $T = 25\,°C$, $35\,°C$ and $55\,°C$. In order to compare the different reactions, the rate constant for crystal growth, k_G, and growth exponent, n_G, were refined to the data as a function of time, according to the Avrami-Erofe'ev expression, $\alpha = 1 - \exp(-(k_G \times t)^{n_G})$, where $0 < \alpha < 1$ is the extent of crystallisation.[12–14]

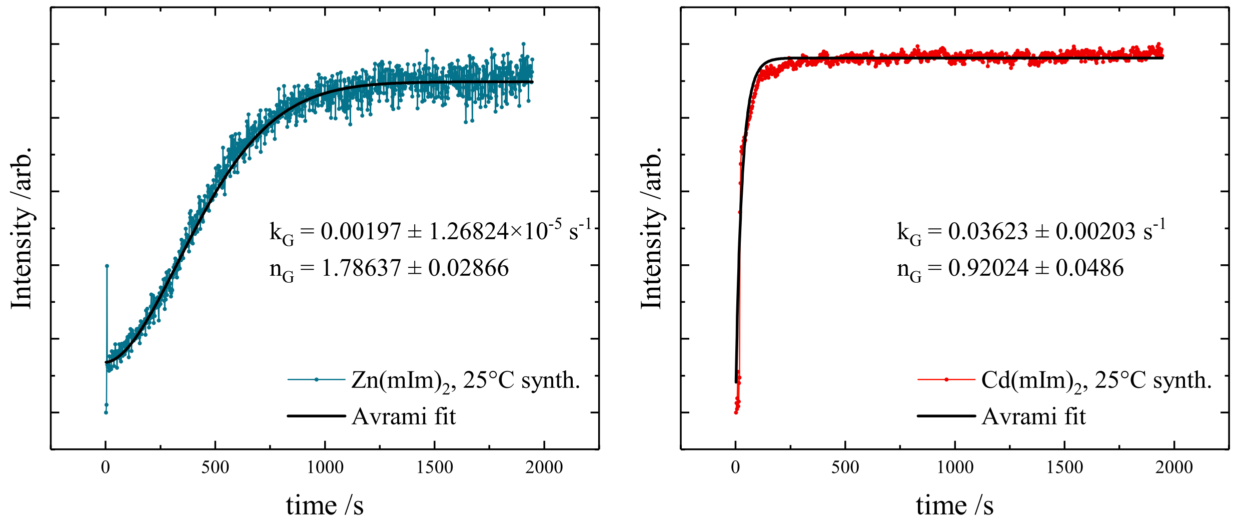

Figure S41: Fit of Avrami-Erofe'ev kinetics to total Bragg peak intensity against reaction time for the formation of Zn-ZIF-8, left, and Cd-ZIF-8, right, at 25 °C.

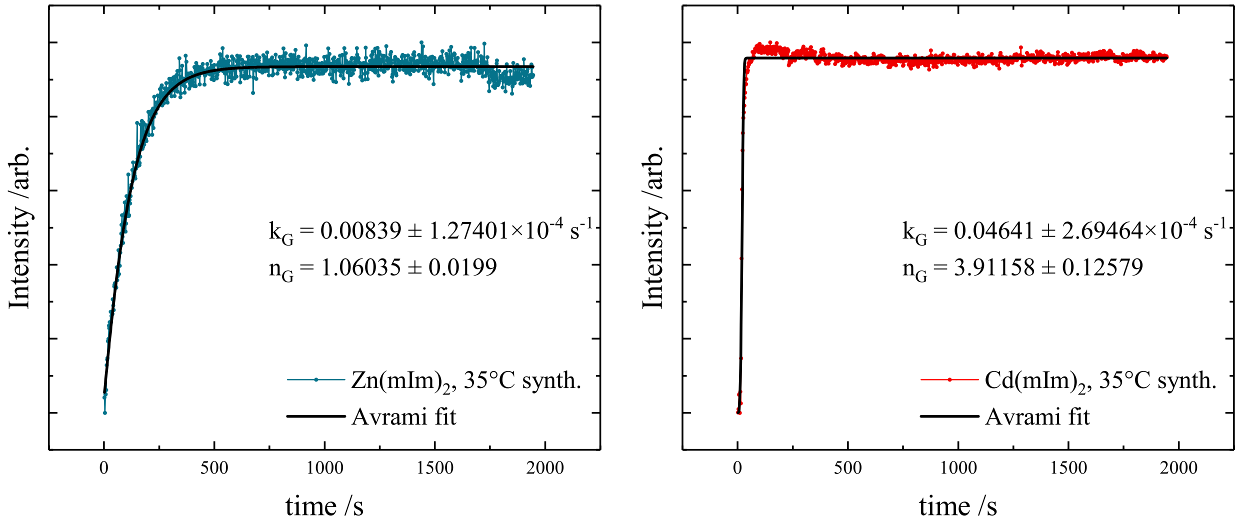

Figure S42: Fit of Avrami-Erofe'ev kinetics to total Bragg peak intensity against reaction time for the formation of Zn-ZIF-8, left, and Cd-ZIF-8, right, at 35 °C.

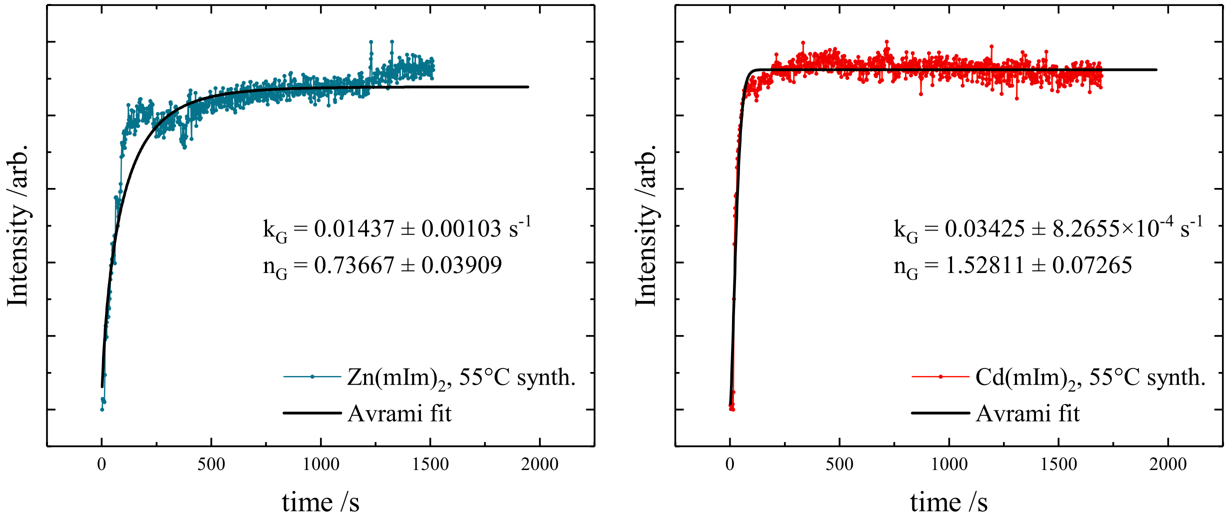

Figure S43: Fit of Avrami-Erofe'ev kinetics to total Bragg peak intensity against reaction time for the formation of Zn-ZIF-8, left, and Cd-ZIF-8, right, at 55 °C.

S10 Bibliography

[1] A. F. Sapnik, H. S. Geddes, E. M. Reynolds, H. H.-M. Yeung and A. L. Goodwin, *Chemical Communications*, 2018, **54**, 9651–9654.

[2] S. P. Thompson, J. E. Parker, J. Marchal, J. Potter, A. Birt, F. Yuan, R. D. Fearn, A. R. Lennie, S. R. Street and C. C. Tang, *Journal of Synchrotron Radiation*, 2011, **18**, 637–648.

[3] J. E. Parker, S. P. Thompson, T. M. Cobb, F. Yuan, J. Potter, A. R. Lennie, S. Alexander, C. J. Tighe, J. A. Darr, J. C. Cockcroft and C. C. Tang, *Journal of Applied Crystallography*, 2011, **44**, 102–110.

[4] M. Drakopoulos, T. Connolley, C. Reinhard, R. Atwood, O. Magdysyuk, N. Vo, M. Hart, L. Connor, B. Humphreys, G. Howell, S. Davies, T. Hill, G. Wilkin, U. Pedersen, A. Foster, N. De Maio, M. Basham, F. Yuan and K. Wanelik, *Journal of Synchrotron Radiation*, 2015, **22**, 828–838.

[5] G. S. Pawley, *Journal of Applied Crystallography*, 1980, **13**, 630–633.

[6] A. A. Coelho, *Journal of Applied Crystallography*, 2018, **51**, 210–218.

[7] P. Thompson, D. E. Cox and J. B. Hastings, *Journal of Applied Crystallography*, 1987, **20**, 79–83.

[8] S. M. Collins, K. E. MacArthur, L. Longley, R. Tovey, M. Benning, C. B. Schönlieb, T. D. Bennett and P. A. Midgley, *APL Materials*, 2019, **7**, 091111.

[9] F. de la Peña, T. Ostasevicius, V. T. Fauske, P. Burdet, E. Prestat, P. Jokubauskas, M. Nord, M. Sarahan, K. E. MacArthur, D. N. Johnstone, J. Taillon, J. Caron, V. Migunov, T. Furnival, A. Eljarrat, S. Mazzucco, T. Aarholt, M. Walls, T. Slater, F. Winkler, B. Martineau, G. Donval, R. McLeod, E. R. Hoglund, I. Alxneit, I. Hjorth, T. Henninen, L. F. Zagonel, A. Garmannslund and 5ht2, *hyperspy/hyperspy: HyperSpy*, 2018.

[10] G. Cliff and G. W. Lorimer, *Journal of Microscopy*, 1975, **103**, 203–207.

[11] K. E. MacArthur, T. J. A. Slater, S. J. Haigh, D. Ozkaya, P. D. Nellist and S. Lozano-Perez, *Microscopy and Microanalysis*, 2016, **22**, 71–81.

[12] M. Avrami, *Journal of Chemical Physics*, 1939, **7**, 1103–1112.

[13] M. Avrami, *The Journal of Chemical Physics*, 1940, **8**, 212–224.

[14] M. Avrami, *The Journal of Chemical Physics*, 1941, **9**, 177–184.
